# Laterality Influences Central Integration of Baroreceptor Afferent Input in Male and Female Sprague Dawley Rats

**DOI:** 10.3389/fphys.2020.00499

**Published:** 2020-05-27

**Authors:** Ibrahim M. Salman, Omar Z. Ameer, Sheridan McMurray, Alessandra S. Giarola, Arun Sridhar, Stephen J. Lewis, Yee-Hsee Hsieh

**Affiliations:** ^1^College of Pharmacy, Alfaisal University, Riyadh, Saudi Arabia; ^2^Division of Pulmonology, Allergy and Immunology, Department of Pediatrics, School of Medicine, Case Western Reserve University, Cleveland, OH, United States; ^3^Department of Disease Biology, Galvani Bioelectronics, Hertfordshire, United Kingdom; ^4^Division of Pulmonary, Critical Care, and Sleep Medicine, Department of Medicine, School of Medicine, Case Western Reserve University, Cleveland, OH, United States

**Keywords:** aortic depressor nerve, baroreflex, baroreceptor afferents, cardiovascular, sexual dimorphism

## Abstract

We explored the effects of baroreceptor afferents laterality and sexual dimorphism on the expression of cardiovascular reflex responses to baroreflex activation in Sprague Dawley (SD) rats. Under urethane anesthesia, rats of either sex (total *n* = 18) were instrumented for left, right and bilateral aortic depressor nerve (ADN) stimulation (1–40 Hz, 0.2 ms, 0.4 mA for 20 s) and measurement of mean arterial pressure (MAP), heart rate (HR) and mesenteric (MVR) and femoral (FVR) vascular resistance. Female rats were matched for the diestrus phase of the estrus cycle. Left, right and bilateral ADN stimulation evoked frequency-dependent drops in MAP, HR, and MVR, and increases in FVR. Irrespective of sex, left and bilateral ADN stimulation as compared to right-sided stimulation mediated greater reflex reductions in MAP, HR, and MVR but not in FVR. In males, reflex bradycardic responses were greater in response to bilateral stimulation relative to both left- and right-sided stimulation. In females, left ADN stimulation evoked the largest increase in FVR. Left and bilateral ADN stimulations evoked greater reductions in MAP and MVR while left-sided stimulation produced larger increases in FVR in females compared with males. All other reflex responses to ADN stimulation were relatively comparable between males and females. These results show a differential baroreflex processing of afferent neurotransmission promoted by left versus right baroreceptor afferent inputs and sexual dimorphism in the expression of baroreflex responses in rats of either sex. Collectively, these data add to our understanding of physiological mechanisms pertaining to baroreflex control in both males and females.

## Introduction

The arterial baroreceptor reflex is a vital short-term control mechanism that maintains blood pressure (BP) within a relatively narrow range of oscillation (Thomas, 2011; Salman, 2016). Activation of this reflex pathway induces inhibition of the sympathetic outflow to the heart and vasculature and upregulates parasympathetic drive to the heart, thereby contributing to reductions in peripheral vascular resistance, cardiac output and ultimately BP (Thomas, 2011; Salman, 2016). Electrical activation of the baroreceptor afferent neurons within the aortic depressor nerve (ADN) in rats represents a reliable method for studying physiological mechanisms pertaining to baroreflex-mediated cardiovascular regulation (Ma et al., 2002; Salman, 2015).

An overwhelming number of studies have investigated the role of baroreceptor afferents in the regulation of cardiovascular function and how the afferent neurotransmission is processed centrally in both normal and disease states (Ma et al., 2002; Salman et al., 2014; Salman et al., 2015; Brognara et al., 2016; Kawada et al., 2018). Most of these studies, however, undertook a unilateral activation of these afferent pathways, without taking into account whether similar findings can be generated when the afferent fibers within the contralateral side are activated or when afferent nerves on both sides are activated simultaneously. Anatomical evidence indicates that the left baroreceptor afferents primarily innervate the aortic arch at the junction region of the left common carotid and left subclavian arteries (Cheng et al., 1997). In contrast, the right baroreceptor afferent bundles, which enter the junction region of the right common carotid artery and the right subclavian artery, synapse onto the lower right common carotid artery and are, therefore, positioned further away from the heart slightly above the aortic arch level (Cheng et al., 1997). It has further been shown that the left ADN tends to have a greater number of axons relative to the right ADN (Cheng et al., 1997). Whether these anatomical differences would translate into a differential left versus right baroreflex-driven cardiovascular response remains unclear, with a limited number of reports supporting lateralization in neural function. A previous *in vitro* study in rabbits reported similar activities in the left and right ADN (Angell-James, 1973); however, if those similarities coincided with comparable efficacy for the activated nerves to evoke physiological responses was not systematically confirmed. Research in dogs seems to suggest a definite laterality in ADN responses, with data indicating a left-sided dominance in the reflex depressor in response to ADN stimulation (Hasimoto and Hirohata, 1936) and superior baroreflex-mediated changes in arterial pressure with left and bilateral sectioning of baroreceptor afferents relative to the elimination of the right afferent neurons (Walgenbach et al., 1981). In rats, on the other hand, such functional evidence is still lacking and, therefore, the first objective of our study was to investigate the effect of laterality on the baroreceptor reflex processing of afferent signals in rats and whether measures of cardiovascular reflexes would differ when stimulation of the afferent neurons is delivered on the left side, right side or bilaterally.

Apart from the laterality issue and potential intra-subjective variance in the processing of left versus right baroreceptor afferent inputs, a substantial literature suggests that autonomic nervous system functioning and its pivotal role in cardiovascular regulation vary in both male and female humans and experimental animals. For instance, female humans, in comparison to males, demonstrate lower heart rate (HR) baroreflex sensitivity (BRS) in response to rapid increases in BP (Abdel-Rahman et al., 1994; Tanaka et al., 2004), but show either similar (Tank et al., 2005), greater (Hogarth et al., 2007) or lower (Christou et al., 2005) sympathetic BRS as assessed by direct measurements of muscle sympathetic nerve activity (SNA). Similarly, BRS in rats appears to be influenced by sex, with mixed reports of both lower (Johnson et al., 2011) and higher (Chen and DiCarlo, 1996) HR baroreflex gain in female rats but similar baroreflex control of SNA to that of males (Chen and DiCarlo, 1996). As far as the baroreceptor afferents are concerned, stimulation of the left ADN in female Sprague Dawley (SD) rats evokes a different compound action potential pattern compared with males, findings which are likely contributed to by lower myelin contents and smaller nerve cross-sectional area in females (Santa Cruz Chavez et al., 2014). Further supporting fundamental neuroanatomical and electrophysiological differences in the aortic baroreceptor afferents between males and females is the expression of a distinct functional subtype of myelinated afferent neurons (Ah-type) within the left ADN of female SD rats, which possibly underlies the enhanced BRS seen in females (Li et al., 2008). Despite these lines of evidence, to date, comprehensive functional studies comparing neural integration of baroreceptor afferent inputs between males and females are still lacking. Therefore, a second objective of this investigation was to determine if baroreflex processing of afferent signals exhibits sexual dimorphism.

## Materials and Methods

### Animals

All studies were carried out in accordance with the National Institutes of Health Guide for the Care and Use of Laboratory Animals. The protocols were approved by the Animal Care and Use Committee of Case Western Reserve University. Adult male and female SD rats (12–20 weeks old, males: 320–460 g and females: 220–260 g, total *n* = 18) were sourced from Envigo, United States. All animals were maintained under a 12-h light/dark cycle and fed with rat chow and water *ad libitum*.

### Female Estrus Cycle Detection

**FIGURE 1:**
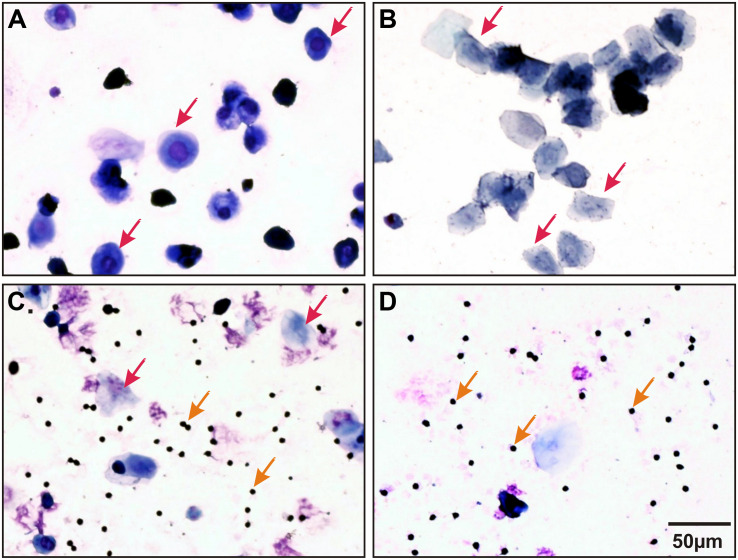
Representative stained (methylene blue, toluidine blue, and hematoxylin) vaginal smears collected from a female Sprague Dawley (SD) rat illustrating all four stages of estrus cycle. **(A)** Proestrus: nucleated epithelial cells (indicated by red arrows); **(B)** Estrus: jagged edged (cornified) non-nucleate cells (indicated by red arrows); **(C)** Metestrus: many leukocytes (indicated by orange arrows) and a few cornified cells (indicated by red arrows) and **(D)** Diestrus: mainly leukocytes (indicated by orange arrows) and a few nucleated cells.

Female rats were matched for the diestrus phase of the estrus cycle where hormonal variations are minimal. Female estrus cycle was tracked as described previously (Marcondes et al., 2002; Yener et al., 2007). Briefly, females were screened in the morning for at least 2 consecutive cycles (8 days) prior to experimentation. Vaginal secretions were collected by minimal insertion of the tip of a plastic pipette filled with 20 μL of saline (0.9% NaCl) into the rat’s vagina followed by flushing of the vaginal contents. Vaginal fluids were then placed on glass slides, fixed, stained (methylene blue, toluidine blue and hematoxylin) and observed under a light microscope to identify different cell types in the sample (Figure 1).

### Surgical Procedures

On the day of neurostimulation experiment, rats were anesthetized with 1.2 g/kg intraperitoneal injection of urethane and maintained with 0.1 ml supplemental intravenous doses of 40% urethane injected into the right femoral vein as required. Core body temperature was maintained using a heating blanket (T/Pump warm water re-circulator, Stryker Medical, MI, United States). Mean arterial blood pressure (MAP) was measured via a cannula inserted into the right femoral artery. Heart rate (HR) was derived from the pulsatile signal of BP. A ventral neck incision was made, and a tracheotomy performed to facilitate spontaneous breathing. A transonic flow probes (TS420 Perivascular Flow Module, Transonic System Inc., New York, NY, United States) were placed around the mesenteric and left femoral arteries to simultaneously measure mesenteric (MBF) and femoral (FBF) blood flow and calculate mesenteric (MVR) and femoral (FVR) vascular resistance, respectively. A 4–6 mm segment of the aortic depressor nerve (ADN) on both the left and right ventral neck region was isolated distal to the point where it entered the superior laryngeal nerve, placed uncut on silver bipolar stimulating electrodes (interelectrode distance of ≈1 mm) and maintained in mineral oil. The bipolar electrodes were placed such that the anode was distal to the cathode, which preferentially blocked distal propagation of action potentials and, thus, favorably directed the current toward the brain (Stauss, 2017). The stimulating electrodes were then connected to a square pulse stimulator (S88 Dual output square pulse stimulator, Grass Technologies Product Group, United States) using a stimulus isolation unit (Grass Instrument Co., Model PSIU6 Photoelectric Stimulus Isolation Unit, Grass Technologies Product Group, United States). The stimulation system delivered a monophasic electrical current and corresponding voltage traces were recorded using a digital oscilloscope (Yokogawa Digital Oscilloscope DL708E, Tokyo, Japan). All recordings were acquired using CED 1401 data acquisition system (Power3A CED 1401, Cambridge Electronic Design Ltd., Cambridge, United Kingdom). At the end of the surgical procedures, animals were allowed to stabilize for 15–30 min before commencing the neurostimulation protocol.

### Experimental Protocol

Left, right and bilateral ADN stimulation was performed at frequencies of 1, 2.5, 5, 10, 20, and 40 Hz, all delivered at 0.4 mA, 0.2 ms for 20 s separated by at least 2 min and peak responses in MAP, HR, MBF, and FBF were continuously recorded. The stimulus intensity and pulse width were determined as described previously (Salman et al., 2017) and the chosen current intensity approximated 0.15 V as displayed on the oscilloscope’s voltage trace. All variables were allowed to return to pre-stimulus baseline levels before the application of the next stimulus. Both the side of stimulation and the order of frequencies were randomized throughout the experiments. At the end of the stimulation protocol, rats were euthanized with an intravenous injection of potassium chloride. All experimental protocols were completed within ≈ 4 h.

### Data Analysis

Data were expressed as mean ± standard error of mean (SEM). All data were analyzed offline using Spike 2 software (Cambridge Electronic Design Ltd., Cambridge, United Kingdom) and GraphPad Prism (GraphPad Prism software v6 Inc., La Jolla, CA, United States).

Raw spike traces were converted into 5-s bins and mean data 40 s prior and 80 s post neurostimulation were plotted against time in seconds. Vascular resistance (VR) was calculated from measures of MAP and regional blood flow (BF) using the following formula, as described previously (Possas et al., 2006):

$$\mathrm{VR}\,\left(\mathrm{mmHg}\cdot \min\cdot {\mathrm{ml}}^{-1}\right)=\frac{\mathrm{MAP}\,\left(\mathrm{mmHg}\right)}{\mathrm{BF}\,\left(\mathrm{ml}\cdot \min^{-1}\right)}$$

Baseline variables were recorded over a 40-s period prior to delivering ADN stimulation and immediately after the fulfilment of the stimulation protocol. Baseline data within each sex and in between males and females were compared using two-way ANOVA followed by Bonferroni’s *post hoc* analysis.

Reflex responses to ADN stimulation were determined by measuring peak changes (both absolute and percentage changes) in MAP, HR, MBF, FBF, MVR, and FVR relative to an immediate 40-s baseline prior to the application of each electrical stimulus. MAP gain, which reflected the immediacy of the BP response, was calculated as the slope of the linear portion of the reflex depressor response generated during the 20-s stimulation period. Within each sex, a two-way ANOVA followed Bonferroni’s correction was used to identify differences in the response variables measured after left, right and bilateral ADN stimulation. Likewise, sex differences in the reflex responses were determined using a two-way ANOVA followed by Bonferroni’s *post hoc* analysis. Significance was defined as *P* ≤ 0.05.

## Results

**FIGURE 2:**
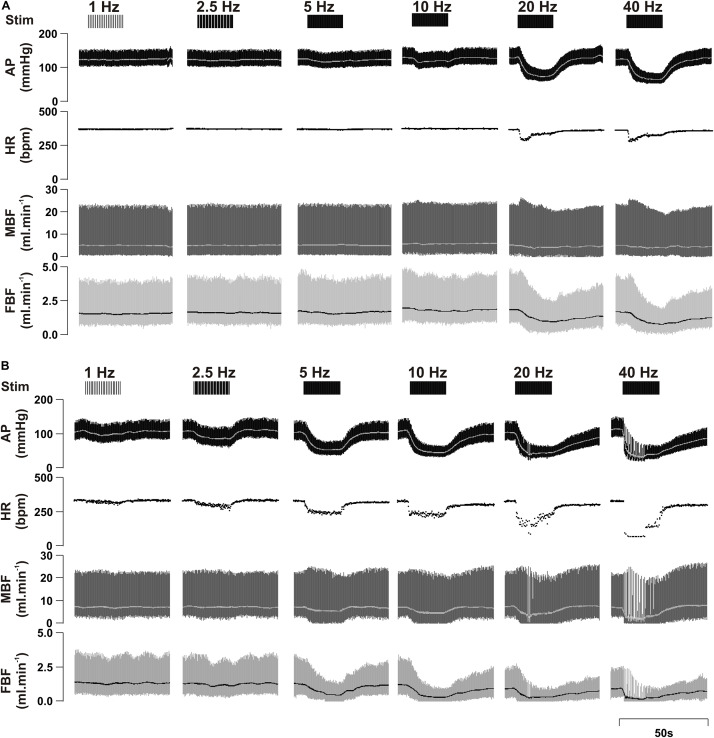
Representative raw data traces showing the effect of left aortic depressor nerve (ADN) stimulation (1, 2.5, 5, 10, 20, and 40 Hz at 0.4 mA, 0.2 ms for 20 s) on cardiovascular parameters measured in urethane-anaesthetized male **(A)** and female **(B)** Sprague Dawley (SD) rats. Simulation (Stim), arterial pressure (AP), heart rate (HR), mesenteric blood flow (MBF) and femoral blood flow (FBF).

Figure 2 provides a sample raw data trace illustrating cardiovascular responses to left ADN stimulation in both male and female SD rats.

Time-trend profiles illustrating the effect of ADN stimulation on recorded cardiovascular parameters (Supplementary Figures S1–S12) and calculated absolute changes in the measured physiological variables (Supplementary Figures S13–S15) are provided in the Online Data Supplement.

### Baseline Hemodynamics

Within the same sex, no baseline hemodynamics differed before ADN stimulation and at the end of the stimulation protocol. Females had lower baseline FBF and higher FVR compared with males. All other hemodynamic variables were similar between males and females (Table 1).

**TABLE 1:** Baseline hemodynamic measures in urethane-anesthetized male and female Sprague Dawley (SD) rats.

Parameter	Male (*n* = 11)		Female (*n* = 6–7)	
		
	Before stimulation	After stimulation	Before stimulation	After stimulation
MAP (mmHg)	1035	984	1134	1092
HR (bpm)	33810	32910	34011	3277
MBF (ml⋅min^–1^)	5.40.4	4.70.3	4.80.2	4.90.4
MVR (mmHg⋅min⋅ml^–1^)	212	222	251	232
FBF (ml⋅min^–1^)	2.480.41	2.050.37	1.010.13^d^	0.720.03^d^
FVR (mmHg⋅min⋅ml^–1^)	5011	6414	12216^d^	1537^d^

*MAP, mean arterial pressure; HR, heart rate; MBF, mesenteric blood flow; MVR, mesenteric vascular resistance; FBF, femoral blood flow; and FVR, femoral vascular resistance. Results are expressed as mean ± SEM, ^*d*^*P* ≤ 0.05 vs. respective male counterpart analyzed by a two-way ANOVA followed by Bonferroni’s *post hoc*.*

### Effect of ADN Stimulation on MAP Responses

**FIGURE 3:**
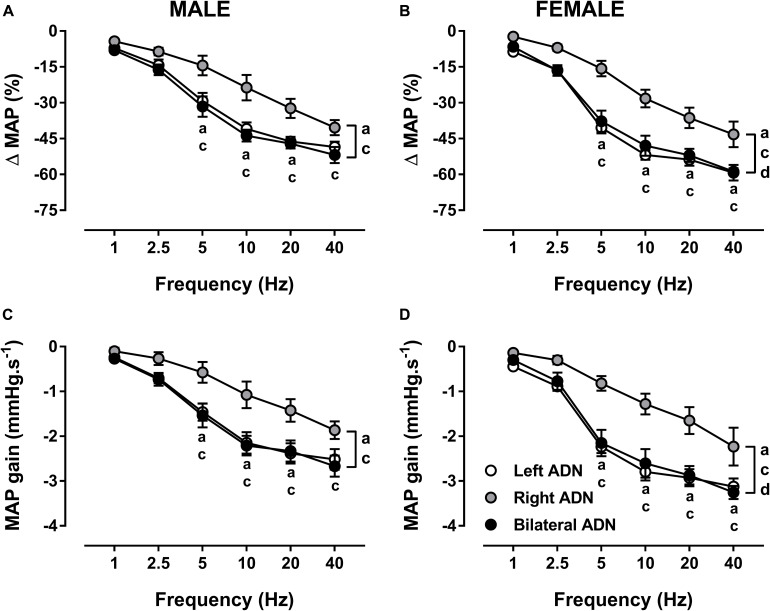
Effects of left, right and bilateral aortic depressor nerve (ADN) stimulation (1–40 Hz, 0.4 mA, 0.2 ms, 20 s) on mean arterial pressure (MAP) **(A,B)** and MAP gain **(C,D)** responses in urethane-anesthetized male **(left panels)** and female **(right panels)** Sprague Dawley (SD) rats (*n* = 7–11). Results are expressed as mean ± SEM. ^a^*P* ≤ 0.05, left vs. right ADN, ^c^*P* ≤ 0.05, right vs. bilateral ADN and ^d^*P* ≤ 0.05, female vs. male for respective left and bilateral ADN analyzed by a two-way ANOVA followed by Bonferroni’s *post hoc*. Note that, in males and females, left and bilateral stimulation evoked greater drops (both overall and at frequencies of 5–40 Hz) in MAP and MAP gain compared to right-sided stimulation. Females’ left and bilateral stimulation displayed greater reflex changes in MAP parameters compared with those of the males’ counterpart.

Irrespective of sex, stimulation of the ADN resulted in frequency-dependent drops (*P* < 0.001) in MAP (Figure 3, Supplementary Figures S1, S7, and S13). Left and bilateral ADN stimulation induced similar reflex reductions in MAP; however, these were significantly greater (*P* < 0.001) compared to reflex depressor responses resulting from stimulation of the right ADN (Figure 3 and Supplementary Figure S13). Likewise, reflex depressor responses, as assessed by MAP gain measures, were evoked at similar rates for both left and bilateral stimulations. In contrast, MAP gain for right-sided stimulation was markedly lower (*P* < 0.001) relative to both left-sided and bilateral stimulation (Figure 3).

Reflex depressor responses and MAP gain for left and bilateral stimulations were greater (*P* < 0.05) in females compared with males. No sex differences in reflex MAP responses were identified with the right-sided stimulation (Figure 3 and Supplementary Figure S13).

### Effect of ADN Stimulation on HR Responses

**FIGURE 4:**
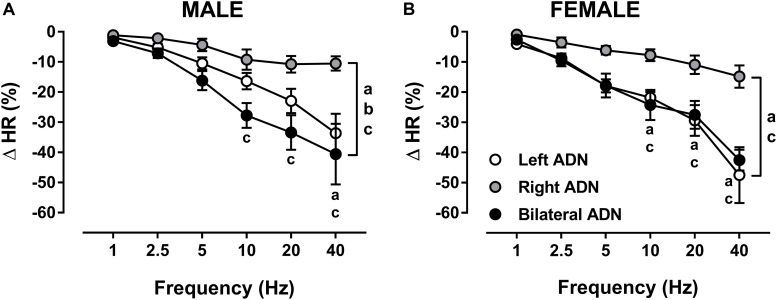
Effects of left, right and bilateral aortic depressor nerve (ADN) stimulation (1–40 Hz, 0.4 mA, 0.2 ms, 20 s) on heart rate (HR) responses in urethane-anesthetized male **(A)** and female **(B)** Sprague Dawley (SD) rats (*n* = 7–11). Results are expressed as mean ± SEM. ^a^*P* ≤ 0.05, left vs. right ADN, ^b^*P* ≤ 0.05, left vs. bilateral ADN and ^c^*P* ≤ 0.05, right vs. bilateral ADN analyzed by a two-way ANOVA followed by Bonferroni’s *post hoc*. Note that bilateral stimulation in males and both left and bilateral stimulation in females evoked the highest drops in HR (both overall and at frequencies above 5 Hz).

Stimulation of the ADN evoked frequency-dependent reductions (*P* < 0.001) in HR in both males and females (Figure 4, Supplementary Figures S2, S8, and S13). In males, bilateral ADN stimulation resulted in the largest drops (*P* < 0.01) in HR compared with HR responses evoked by both left and right ADN stimulations. Reflex bradycardia in response to left ADN stimulation were also greater (*P* < 0.05) compared with right ADN stimulation. Reflex bradycardic responses in females mimicked those of their corresponding reflex depressor responses, showing parallel reflex reductions in HR in response to left and bilateral ADN stimulation. Right-side-mediated reflex bradycardic responses, on the other hand, were lower (*P* < 0.05) relative to those evoked by left-sided and bilateral stimulations (Figure 4 and Supplementary Figure S13).

Unlike the reflex depressor response, the reflex bradycardic responses to left, right and bilateral ADN stimulation compared very closely in both males and females (Figure 4 and Supplementary Figure S13).

### Effect of ADN Stimulation on Mesenteric Vascular Responses

**FIGURE 5:**
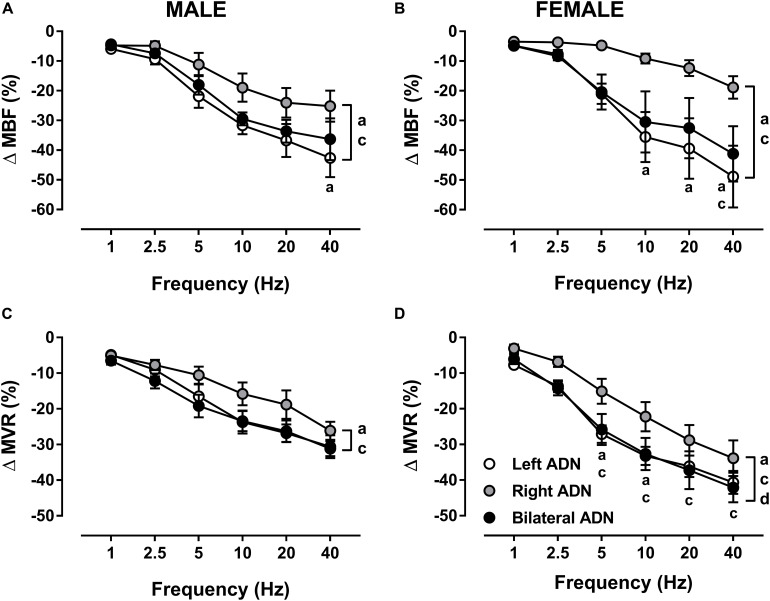
Effects of left, right and bilateral aortic depressor nerve (ADN) stimulation (1–40 Hz, 0.4 mA, 0.2 ms, 20 s) on mesenteric blood flow (MBF) **(A,B)** and mesenteric vascular resistance (MVR) **(C,D)** responses in urethane-anesthetized male **(left panels)** and female **(right panels)** Sprague Dawley (SD) rats (*n* = 6–9). Results are expressed as mean ± SEM. ^a^*P* ≤ 0.05, left vs. right ADN, ^c^*P* ≤ 0.05, right vs. bilateral ADN and ^d^*P* ≤ 0.05, female vs. male for respective left and bilateral ADN analyzed by a two-way ANOVA followed by Bonferroni’s *post hoc*. Note that, in both males and females, left and bilateral stimulation evoked the greatest drops (both overall and variably at frequencies ranging between 5 and 40 Hz) in MBF and MVR. Females’ left and bilateral stimulation showed greater reflex reductions in MVR compared with those of the males’ counterpart.

In both males and females, stimulation of the ADN evoked frequency-dependent drops (*P* < 0.001) in MBF (Figure 5, Supplementary Figures S3, S9, and S14) and MVR (Figure 5, Supplementary Figures S4, S10, and S14). Like MAP responses, left and bilateral ADN stimulation induced similar reflex drops in MBF and MVR, with these responses being markedly greater (*P* < 0.05) as compared with MBF and MVR reductions due to stimulation of the right ADN. Reflex MBF measures did not differ between male and females. Reflex MVR responses to left and bilateral, but not right, ADN stimulation, by contrast, were markedly greater (*P* < 0.01) in females compared with males (Figure 5). Absolute changes in MBF and MVR compared very closely with percentage data (Supplementary Figure S14).

### Effect of ADN Stimulation on Femoral Vascular Responses

**FIGURE 6:**
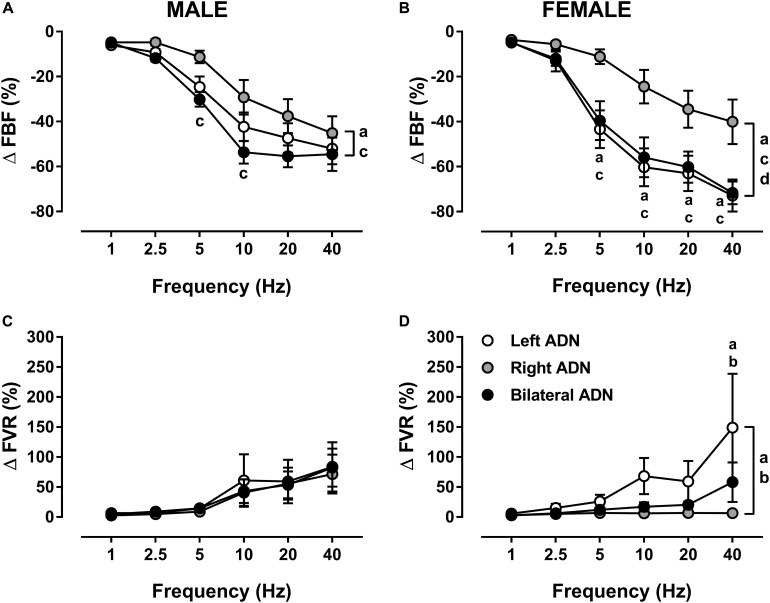
Effects of left, right and bilateral aortic depressor nerve (ADN) stimulation (1–40 Hz, 0.4 mA, 0.2 ms, 20 s) on femoral blood flow (FBF) **(A,B)** and femoral vascular resistance (FVR) **(C,D)** responses in urethane-anesthetized male **(left panels)** and female **(right panels)** Sprague Dawley (SD) rats (*n* = 7–11). Results are expressed as mean ± SEM. ^a^*P* ≤ 0.05, left vs. right ADN, ^b^*P* ≤ 0.05, left vs. bilateral ADN, ^c^*P* ≤ 0.05, right vs. bilateral ADN and ^d^*P* ≤ 0.05, female vs. male for respective left ADN analyzed by a two-way ANOVA followed by Bonferroni’s *post hoc*. Note that, in both males and females, left and bilateral stimulation evoked the greatest drops (both overall and variably at frequencies ranging between 5 and 40 Hz) in FBF. Reflex FVR responses did not differ in the males but, in females, measures were the highest for the left-sided stimulation (both overall and at a frequency of 40 Hz).

Stimulation of the ADN produced frequency-dependent decreases (*P* < 0.001) in FBF in both males and females (Figure 6, Supplementary Figures S5, S11, and S15). Reflex reductions (%) in FBF were not different between left and bilateral stimulations but were lower (*P* < 0.05) for right-sided stimulation as compared to both left and bilateral ADN stimulation (Figure 6). These differences were not evident in males when results were expressed as absolute change (Supplementary Figure S15). Reflex reductions (%) in FBF mediated by left-sided, but not right-sided or bilateral, stimulation were greater (*P* < 0.01) in females relative to males (Figure 6 and Supplementary Figure S15). This sex difference was not evident when data were reported in absolute terms (Supplementary Figure S15).

Irrespective of sex, ADN stimulation evoked frequency-dependent increases (*P* < 0.05) in FVR (Figure 6, Supplementary Figures S6, S12, and S15). In males, these reflex increases in FVR were not different when stimulation of the ADN was delivered to the left side, right side or bilaterally. In females, however, reflex increases in FVR due to left ADN stimulation were greater (*P* < 0.01) compared with those of the right-sided stimulation. Females also had almost greater (*P* = 0.06) left-sided reflex FVR responses than those evoked by the bilateral stimulation; hence, in females, right-sided and bilateral ADN stimulations mediated similar reflex changes in FVR (Figure 6). When reported as a percentage, reflex changes in FVR due to left, right and bilateral ADN stimulation did not differ between males and females (Figure 6). When reported in absolute values, reflex increases in FVR evoked by only left-sided stimulation were greater (*P* < 0.001) in females (Supplementary Figure S15).

## Discussion

The principal novel aspects of this study are (1) a superior baroreflex processing of afferent neurotransmission evoked by left aortic baroreceptors, which was primarily evidenced by greater baroreflex-mediated reductions in BP relative to activation of the right afferent fibers in rats of either sex, (2) neurostimulation of baroreceptor afferent input using bilateral ADN stimulation does not offer additive reflex depressor effects and is not superior to left ADN stimulation, (3) in males, but not female, reflex bradycardic responses appear to exhibit an additive pattern when ADN stimulation is delivered bilaterally as opposed to either right or left ADN stimulation, and (4) baroreflex processing of the left or bilateral baroreceptor afferent input in females evokes higher reflex depressor responses compared with males. Together, this indicates that laterality as well as sexual dimorphism influence central integration of left versus right baroreceptor afferent inputs in male and female normotensive rats.

In both male and female SD rats, depressor responses due to stimulation of the left ADN were always greater compared to those of the right-sided stimulation. These findings indicate that baroreflex integration of left versus right baroreceptor afferent input is differentially expressed in both males and females. The mechanisms underlying these effects remain to be investigated but these may potentially relate to inherent left versus right differences within the primary components of the baroreflex circuit including (1) the afferent arm and the established anatomical and morphological dissimilarities in the afferent fibers [e.g. the closer positioning of the left afferent innervation to the heart and their relatively higher number of sensory fibers compared with the right baroreceptor afferents (Cheng et al., 1997)], (2) the central component (e.g. functional or anatomical differences in the key medullary nuclei processing baroreflex signals namely the nucleus tractus solitarius, NTS; caudal ventrolateral medulla, CVLM and rostral ventrolateral medulla, RVLM), and/or (3) the efferent portion (e.g. functional or anatomical differences in the efferent fibers and impulse conduction properties). Although in a different rat strain and different target nerve, left-sided cervical vagus nerve stimulation also appears to elicit more pronounced cardiorespiratory responses than right-sided stimulation in stroke-prone spontaneously hypertensive rats (Stauss, 2017). It is, therefore, possible that laterality, at least in rats, extends to a range of neuronal pathways within the neurocircuitry.

Of note, bilateral stimulation of the ADN in both sexes was as effective as left ADN stimulation in eliciting reflex reductions in BP and no additive reflex depressor due to bilateral stimulation was observed. These results indicate a lack of cooperativity between the left and right baroreceptor afferent neurons in mediating reflex reductions in BP. One possible explanation for these findings is that in the setting of simultaneous activation of left and right baroafferents, left-sided neuronal traffic may have overridden or silenced central integration of afferent signals traveling within the right baroafferent fibers, hence contributing comparable reflex responses to both left and bilateral stimulations. Although further experiments are required to validate this assumption, the patterns of HR and vascular responses discussed below may, at least partly, provide clues for autonomic control mechanisms underlying the absence of cooperativity in driving the BP response to bilateral ADN stimulation.

The reflex bradycardic response to baroreflex activation is largely mediated by changes in the efferent parasympathetic (vagal) neuronal discharge (Stornetta et al., 1987; De Paula et al., 1999). In males and females, reflex bradycardic responses due to stimulation of the left ADN were markedly greater compared to those triggered by the right-sided stimulation, findings that are consistent with the observed reflex BP responses in rats of either sex. These data suggest that baroreflex processing of left versus right baroreceptor afferent neurotransmission contributes differential vagal outflow to the heart. The data also suggest that differential BP responses evoked by the left versus right afferent fibers are likely contributed to by parallel reflex changes in HR. Intriguingly, the reflex bradycardic response in male rats was additive when bilateral stimulation was performed, a finding which was not observed in female rats. These results indicate that, at least in males, a possible cooperativity between left and right baroreceptor afferents in modulating reflex vagal outflow to the heart and ultimately HR exists. This cooperativity, however, does not seem to contribute toward the overall reflex depressor response evoked by bilateral stimulation of the ADN, hence indicating that lack of cooperativity in mediating the overall BP response is not driven by corresponding reflex changes in HR.

A major contributor to overall BP control is changes in VR. In mathematical terms, changes in VR within a particular vascular bed are indirectly determined by changes in BP and local BF (Carroll, 2001). Parallel changes in BP and BF contribute to no changes in VR. When changes in BP levels exceed that of BF, an increase in VR is observed, hence reflecting an “active vasoconstriction.” In contrast, greater changes in BF relative to BP contribute reductions in VR, thus reflecting an “active vasodilation.” In neurovascular terms, the baroreflex-mediated vasodilation and fall in BP are determined by both the magnitude of inhibition of SNA and the vascular response to sympathetic withdrawal (De Paula et al., 1999; Salgado et al., 2007). Our experiments sought to record blood flow from two vascular beds (mesenteric and femoral) in order to assess whether the baroreflex would mediate comparable changes in VR across different vascular beds.

In general, reflex BF changes recorded from the mesenteric and femoral vascular beds of male and female rats paralleled those of the reflex depressor response, findings which were not unexpected as changes in organ blood flow are directly proportional to changes in BP (Carroll, 2001). Changes in VR within the same sex, however, differed between the mesenteric and femoral blood vessels. Consistent with previous studies (Possas et al., 2006; Salgado et al., 2007), ADN stimulation evoked frequency-dependent reductions in MVR. Similar to the reflex depressor response, left ADN stimulation evoked greater reductions in calculated MVR compared to the right-sided stimulation and equipotent responses compared to bilateral stimulation. These data suggest that baroreflex integration of left versus right baroreceptor afferents potentially contributes differential sympathetic outflow to the mesenteric vasculature. The data further indicate that the differential BP responses evoked by the left-sided versus right-sided stimulation are not only driven by corresponding changes in the reflex bradycardic response but are also likely contributed to by parallel reductions in MVR. Consistent with the reflex depressor response, cooperativity between the right and left baroreceptor afferents in eliciting reflex MVR responses was not observed when stimulation of the ADN was delivered bilaterally. It is, therefore, possible that the lack of an additive reflex depressor response to bilateral ADN stimulation is driven to a larger extent by changes in VR as (1) the additive reflex bradycardic response to bilateral stimulation, at least in males, did not drive larger depressor responses as compared to left-sided stimulation and (2) only changes in reflex MVR responses matched those of BP. Collectively, these findings reaffirms the substantial contribution of MVR to overall control of peripheral VR (Rowell et al., 1972; Christensen and Mulvany, 1993) and highlights, though indirectly, a critical role for mesenteric vascular sympathetic input in overall BP control.

In contrast to MVR, the reflex FVR, which is reflective of changes in hindquarter resistance, was increased in response to ADN stimulation in both male and female rats. While these findings are unprecedented, we believe that the secondary increase in FVR may represent a compensatory “reactive vasoconstriction” mechanism coming into play to counteract the drops in BP in response to baroreflex activation. This view raises the possibility that activation of the baroreflex does not inhibit all the preganglionic motor nerves supplying postganglionic nerves in the lumbar trunk innervating the femoral vascular bed and rather increases the activity of a subpopulation of postganglionic lumbar sympathetic vasoconstrictor fibers. Indeed, it has been suggested that sympathetic vasomotor neurons are topographically distributed within the RVLM (McAllen and May, 1994) and therefore their neuromodulation may selectively alter regional VR. An additional possibility is that co-activation of afferent pathways causing reflex increases in sympathetic activity such as aortic chemoreceptors (Brophy et al., 1999) and/or stimulus spread to other afferents including the superior and recurrent laryngeal nerves, vagus nerve, cervical sympathetic chain and carotid body may have preferentially influenced FVR in our experiments. Alternatively, the acute reflex reduction in BP may concomitantly modulate non-baroreceptor afferent pathways, thereby upregulating sympathetic drive to the femoral vasculature. Taken together with MVR responses, these data indicate that, within the same sex, baroreflex-driven changes in VR are selectively regulated across different vascular beds. We previously showed that, in the iliac vascular bed of pentobarbital-anesthetized SD rats, hindquarter resistance was markedly reduced in response to left ADN stimulation (Possas et al., 2006). When measured in the inferior abdominal aorta, hindquarter resistance was also blunted in response to left ADN stimulation in conscious SHRs (Salgado et al., 2007). The precise mechanism underlying these contrasting findings cannot be deduced from the present study but it may relate to differences in (1) the investigated vascular bed from which hindquarter resistance was measured, or (2) the nerve stimulation protocol (low charge injection in this study versus ultrahigh charge injection in previous studies).

In males, reflex FVR responses did not differ in relation to the stimulation side. In females, however, the left-sided stimulation evoked the greatest reflex increase in FVR. Of note is also the finding that baseline and reflex FVR responses to left ADN stimulation were markedly greater in female rats compared to males. These effects are most likely due to smaller diameter femoral arteries in females, which offers greater resistance to flow (Carroll, 2001; Safar and Struijker-Boudier, 2010).

Comparisons of baroreflex efficacy in males and females have yielded inconsistent results, in part due to differences in direction of change in BP, the type and speed of the stimulus, measurements of the threshold, range or gain of baroreflex function, and method of evoking baroreflexes. In rats, measurement of baroreflexes by the sequence method shows no differences in baroreflex gain (Johnson et al., 2011), but measurements using the modified Oxford method report higher baroreflex gain in female rats compared to males (Chen and DiCarlo, 1996). These observations suggest that while negligible differences exist with arterial pressure near basal levels, when the larger range of the reflex is examined, sex differences emerge. Indeed, exploring the whole frequency range in our experiments strongly indicated that sexual dimorphism in baroreflex function exists and female rats expressed larger and more immediate reflex reductions in BP to left and bilateral ADN stimulation compared with males. These effects are unlikely driven by changes in HR as HR responses did not differ between males and females. Instead, reductions in MVR may have been responsible for the larger depressor responses observed in the females as MVR reflex measures evoked by both left and bilateral ADN stimulation were also larger in females relative to males. It remains to be elucidated, however, if these differences are due to inherent neuroanatomical differences in the baroreceptor afferent fibers between male and female rats or merely relate to a sex hormones influence. Indeed, baroreceptor afferent fibers display lower myelin content and exhibit smaller nerve cross-sectional area in female SD rats compared to males (Santa Cruz Chavez et al., 2014). Further, a subset of distinct myelinated (Ah-type) afferent fibers within the ADN has been identified in female SD rats but not in males (Li et al., 2008), suggesting a possible link between afferent nerve morphology and sexual dimorphism in the expression of baroreflex-mediated cardiovascular effects. It is, therefore, possible that both smaller nerve cross-sectional area, which would likely offer a lower resistance to action potential propagation, and the additional input from Ah-type myelinated fibers in females could have driven their enhanced vascular responses to ADN stimulation. In relation to sex hormones, another potential underlying cause for conflicting findings on sex differences in baroreflex function across different studies, estrogen is known to enhance baroreflex efficacy through a direct action on afferent nerves (Li et al., 2008) and exogenous estrogen supplementation restores baroreceptor afferents excitability in ovariectomized rats (Qiao et al., 2009). It has further been shown that BRS measured during the proestrus phase of the estrus cycle, a phase where levels of gonadal hormones are elevated, is higher relative to that of the estrus or diestrus phase, and with ovariectomy abolishing cycle-induced variation in BRS (Goldman et al., 2009). Similar increases in BRS were identified during the mid-luteal phase of women’s menstrual cycle, when estrogen and progesterone levels are the highest (Minson et al., 2000). To minimize hormonal contribution to baroreflex modulation in these experiments, only female rats during the diestrus phase of the estrus cycle, where peak changes in sex hormones are unattainable, were used. This does not, however, completely discount the possibility of a sex hormone influence as sex differences in the measured variables were still detectable. As such, ovariectomy studies in rats are needed to fully ascertain the contribution of gonadal hormones to sexual dimorphism in the expression of baroreflex function in both males and females.

### Limitations

A limitation of our study is that the left and right carotid sinus nerves (CSNs), which are also involved in baroreflex control, were left intact. It is, therefore, possible that these sensory inputs could have partly buffered the recorded hemodynamic responses to ADN stimulation, and that denervation of those afferents could have contributed larger reflex cardiovascular responses. However, we believe that the assessment of aortic baroreflex function in the presence of intact left and right CSN is more reflective of normal physiological conditions. Furthermore, it has been shown that baroreflex responses to unilateral stimulation of the ADN did not significantly differ in the presence or absence of the contralateral ADN and both CSNs (Fan et al., 1996).

The ADN in rats carries a relatively pure population of barosensitive afferent neurons (Sapru and Krieger, 1977; Sapru et al., 1981; Numao et al., 1985; Kobayashi et al., 1999), which allows for selective investigation of baroreceptor reflex function in this species. Direct electrical stimulation of these afferent fibers enables a defined control of the afferent input into the central nervous system (CNS) and, therefore, assessment of the central portion of the baroreflex arc (Ma et al., 2002; Salman, 2015). A limitation of this method, however, is that the synchronous continuous activation of essentially all afferents within the ADN differs from the physiological pattern of action potential firing, which is in phase with the arterial pressure pulse and is dependent on the recruitment pattern of individual baroreceptor fibers in relation to BP levels, the pressure thresholds of individual fibers, and fiber type (Andresen et al., 1978; Chapleau et al., 1988). A major advantage, on the other hand, is the ability to alter fibers recruitment within a particular nerve by modifying the total charge delivered to the nerve fibers, which is a function of pulse intensity (voltage/current), pulse frequency and pulse duration. Nonetheless, most of the studies in the field of neurostimulation employ supramaximal stimulation currents/voltages (De Paula et al., 1999; Salgado et al., 2007; Huber and Schreihofer, 2010; Salman et al., 2014; Salman et al., 2015) to alter recruitment patterns of A and C fibers when studying functional aspects of nerves. While this approach defines the whole range of responses a nerve can elicit, generated outcomes are far from physiological and have limited translatability into clinical practice. The present experiments utilized a low charge injection of fixed pulse width of 0.2 ms and pulse intensity of 0.4 mA to, at least partially, overcome possible procedure limitations and enable low energy consumption for neuromodulation and potentially maintain the integrity of the activated neuronal units. The choice of stimulation parameters was based on a pilot study in which we were able to demonstrate “no added benefit” of pulse amplitudes beyond 0.4 mA in terms of driving further reflex depressor responses to ADN stimulation (Salman et al., 2017). The pulse frequency, on the other hand, was varied over a wide range to alter recruitment of myelinated A-fiber versus unmyelinated C-fiber afferents within the ADN and thus explore the full frequency-response relationship. Essentially, low-frequency (<10 Hz) stimulation predominantly triggers C-fiber type, while high-frequency (>10 Hz) stimulation appears to additionally recruit A-fiber type (Fan and Andresen, 1998; Fan et al., 1999). Interestingly, differential left versus right reflex depressor responses were detectable below and above 10 Hz of activation frequencies, suggesting that reflex BP changes are independent of the afferent fiber type recruited during the electrical stimulation. In contrast, changes in HR evoked by left, right and bilateral ADN stimulation were similar within each sex at low frequencies but were significantly enhanced for the left and bilateral stimulation at supramaximal frequencies. These results suggest that reflex vagal outflow and the resulting cardiac response to activation of C-fiber afferents is independent of the stimulation side and an additional input from A-fiber afferents in both sex and Ah-type fibers in females at higher frequencies of stimulation enhances the reflex bradycardic response to a greater extent within the left afferent pathway as compared to the right.

Another limitation of the study is that responses in only target organs (heart and vasculature) receiving efferent sympathetic and/or parasympathetic inputs were recorded and that no direct measure of accompanying efferent nerve activity was assessed. It is, therefore, not possible to ascertain if differences in reflex cardiovascular responses were related to triggered changes in autonomic outflows or were otherwise due to altered vascular reactivity to the neural input or both. It is well established that baroreceptor afferent stimulation reflexively activates parasympathetic outflow to the heart while inhibiting sympathetic nerve activity to the heart and a number of vascular beds (Thomas, 2011; Salman, 2016). For instance, stimulation of the ADN has been shown to evoke a reflex reduction in both splanchnic and lumbar sympathetic nerve activity (Huber and Schreihofer, 2010; Simmonds et al., 2014) supplying the mesenteric and femoral vasculature, respectively. While this evidence strongly supports our finding of a reflex drop in MVR in response to ADN stimulation, our observation of baroreflex-mediated increases in FVR in both males and females argues against this notion, suggesting a need for further studies to explore mechanisms underlying neuro-effector interactions.

Lastly, the lack of control experiments, in which the ADN is cut and the neurostimulation protocol is delivered to the central cut end, remains a limitation of the present study as these experiments would unequivocally prove if possible off-target effects due to confounding variables such as stimulus spread or the lack thereof exist.

### Perspectives

Despite substantial literature on the role of the baroreceptor reflex in controlling cardiovascular homeostasis, our knowledge of physiological mechanisms relating to its functioning is still an area of active investigation. The results of the present study provide, for the first time, a direct physiological insight into the role of laterality of baroreceptor afferent fibers and sexual dimorphism in modulating the central processing of baroreceptor afferent input and reflex cardiovascular functions. These data, therefore, add to our understanding of baroreflex mechanisms operating under disease-free conditions and their contribution to cardiovascular regulation in both males and females.

Functional mapping of the neural network is imperative and, irrespective of findings from the present study, reporting the presence or absence of lateralization in neural function is equally important and is a key to fully characterizing various bodily functions. In the presence of scarce functional evidence that supports lateralization we strongly believe that our current study provides novel data to show that apparent similarities in the activity of the left and right ADNs in rats do not correspond to comparable efficacy of these inputs in evoking reflex cardiovascular responses, hence highlighting the importance of studying both attributes (activity and efficacy). Our results further demonstrate that laterality in neural functions is highly reproducible in both sexes for multiple measures and stimulus frequencies. Such observations are crucial when designing experiments examining the ADN, which may randomly select one side or the other. Future studies must clearly state which side was used as side selection can change the answer to the formulated research question.

Although beyond the scope of the present study, future studies will also need to determine the relative necessity of the left and right ADNs for rats. The two existing studies in rats that serially remove one side or the other and show the importance of both sides do not control for which side was eliminated first (Pickering et al., 2008; Ishii et al., 2015), which likely leaves a false impression of comparable relative importance. To this end, baroreflex bradycardia seems to be more dependent on both left and right ADN than left and right CSNs, while reflex sympathoinhibition is dependent on all four baroafferents, with the stimulation of each single side resulting in a robust sympathoinhibition irrespective of the denervation sequence (Pickering et al., 2008; Ishii et al., 2015). Indeed, to date, no systematic examination of contributions from sectioning the left or right side was performed in rats despite reports of differences in the left and right ADN in terms of sites of innervation of the aorta, relative ease in locating the nerves on the left side, and total number of fibers within the afferent neurons.

## Data Availability Statement

All datasets generated for this study are included in the article/Supplementary Material.

## Ethics Statement

The animal study was reviewed and approved by the Animal Care and Use Committee of Case Western Reserve University.

## Author Contributions

IS contributed to design of the research, performed the experiments, analyzed the data, interpreted results of the experiments, prepared the figures, drafted the manuscript and approved and submitted final version of the manuscript. OA assisted in the undertaking of the experiments and approved final version of the manuscript. SM, AG, AS, SL, and Y-HH contributed to the design of the research and revised and approved final version of the manuscript.

## Conflict of Interest

SM, AG, and AS are based in the Department of Disease Biology of Galvani Bioelectronics (United Kingdom), with AS being the Head of the Department and SM and AG being the Project Managers. The remaining authors declare that the research was conducted in the absence of any commercial or financial relationships that could be construed as a potential conflict of interest.
