# Supplementary material for: Laterality Influences Central Integration of Baroreceptor Afferent Input in Male and Female Sprague Dawley Rats
Source: Front Physiol. 2020 May 27;11:499. doi: 10.3389/fphys.2020.00499 (PMC7269127; doi:10.3389/fphys.2020.00499)
Supplement: Supplementary file 1 [file Data_Sheet_1.pdf]

Laterality Influences Central Integration of Baroreceptor Afferent Input in Male and Female Sprague Dawley Rats

Online Data Supplement

Ibrahim M. Salman [1,2*], Omar Z. Ameer [1], Sheridan McMurray [3], Arun Sridhar [3], Alessandra Giarola [3], Stephen J. Lewis [2] and Yee-Hsee Hsieh [4]

[1] College of Pharmacy, Alfaisal University, Riyadh, Saudi Arabia

[2] Division of Pulmonology, Allergy and Immunology, Department of Pediatrics, School of Medicine, Case Western Reserve University, Cleveland, Ohio, United States

[3] Department of Disease Biology, Galvani Bioelectronics, Hertfordshire, United Kingdom

[4] Division of Pulmonary, Critical Care, and Sleep Medicine, Department of Medicine, School of Medicine, Case Western Reserve University, Cleveland, Ohio, USA

Running title: laterality and baroreceptor reflex

Number of figures: 15

*** Correspondence**

Ibrahim M. Salman, PhD

Address: Department of Pediatrics, Biomedical Research Building, School of Medicine, Case Western Reserve University, 2109 Adelbert Road, Cleveland, Ohio 44106, United States

Email: ibrahim.salman@case.edu

Phone: +121663683438

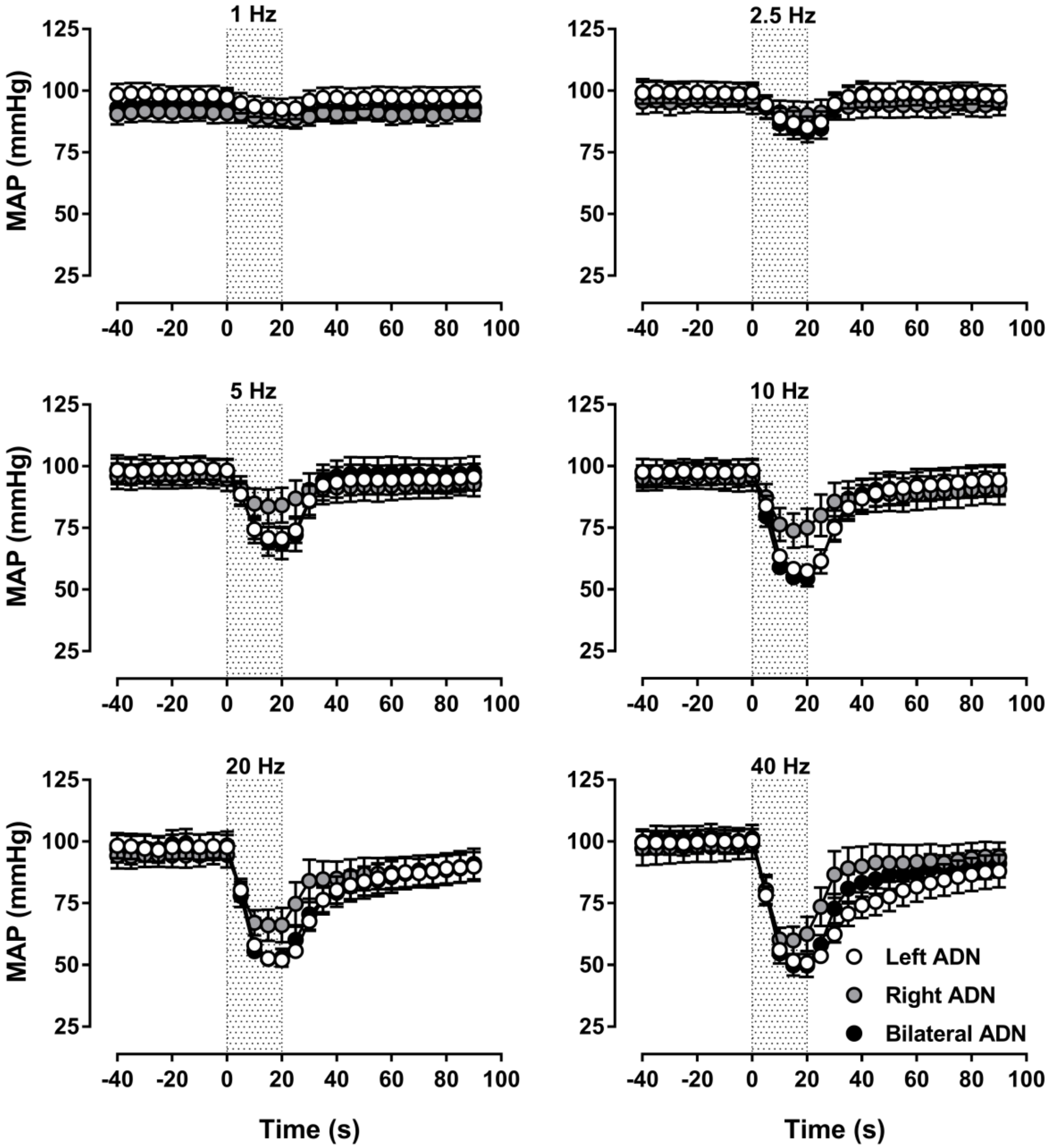

FIGURE S1: Time trend profile for the effects of left, right and bilateral aortic depressor nerve (ADN) stimulation (1–40 Hz, 0.4 mA, 0.2 ms, 20s) on mean arterial pressure (MAP) responses in a urethane-anesthetized male Sprague Dawley (SD) rats (*n*=7–11). Results are expressed as mean ± SEM.

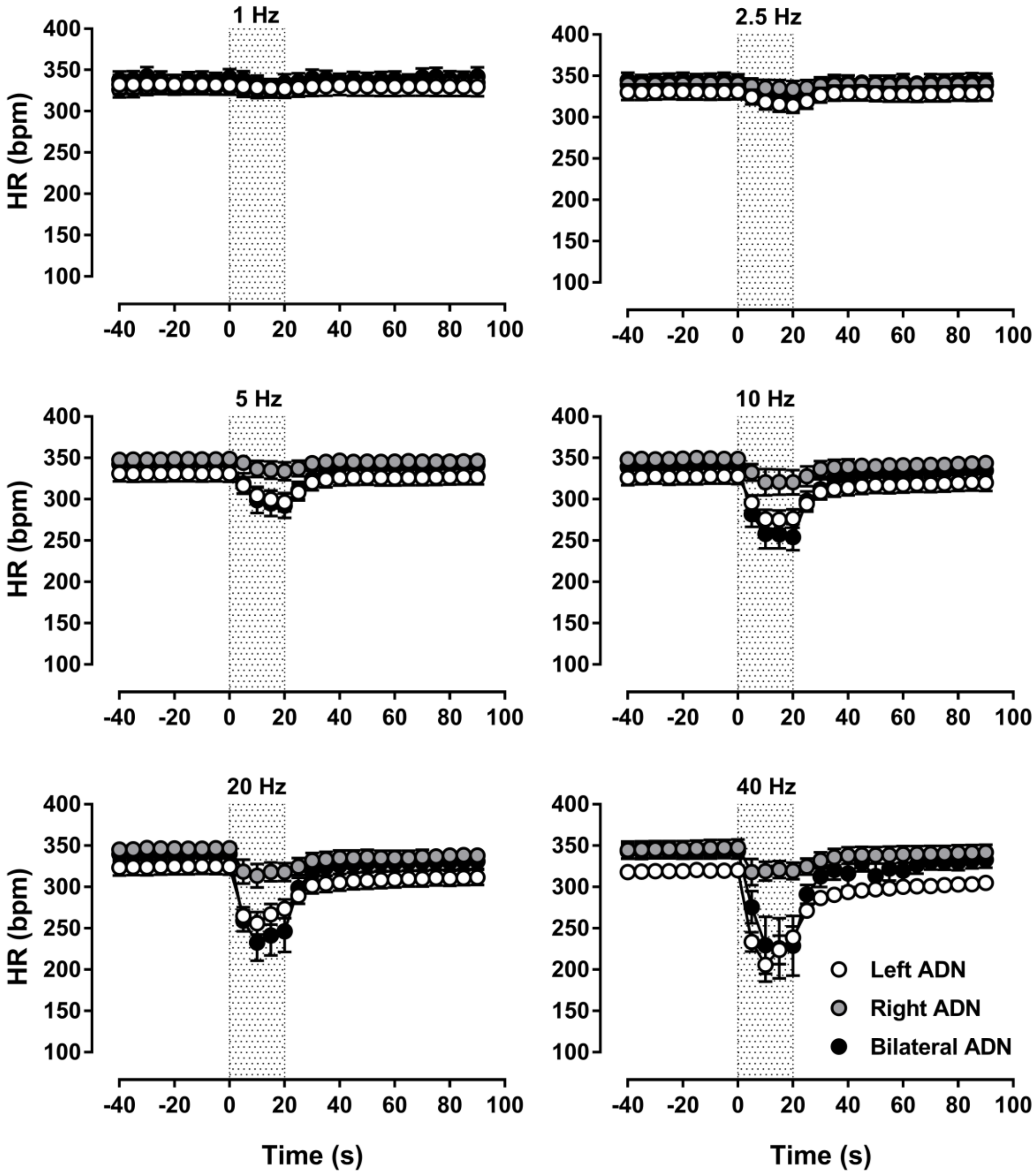

FIGURE S2: Time trend profile for the effects of left, right and bilateral aortic depressor nerve (ADN) stimulation (1–40 Hz, 0.4 mA, 0.2 ms, 20s) on heart rate (HR) responses in a urethane-anesthetized male Sprague Dawley (SD) rats (*n*=7–11). Results are expressed as mean ± SEM.

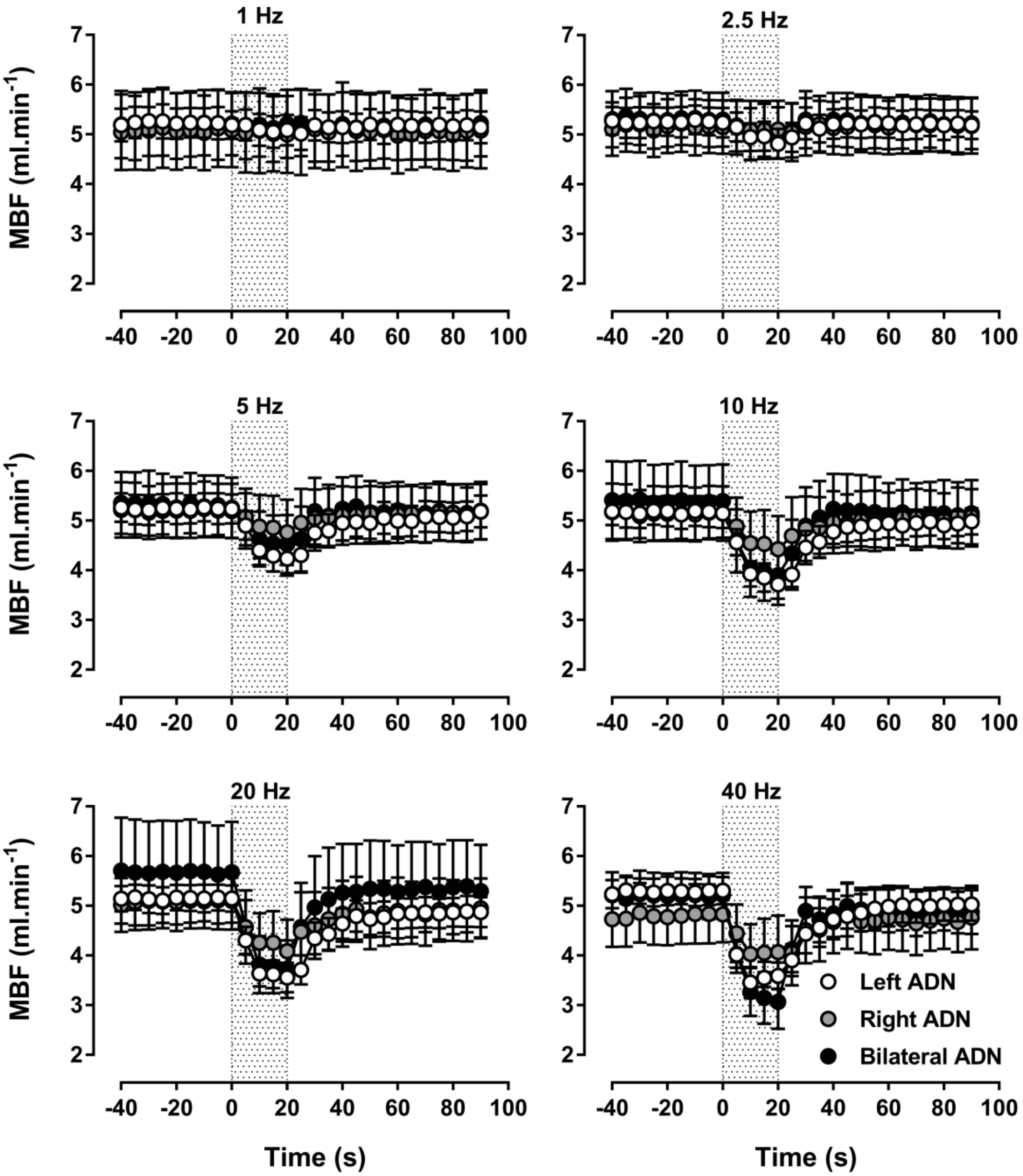

FIGURE S3: Time trend profile for the effects of left, right and bilateral aortic depressor nerve (ADN) stimulation (1–40 Hz, 0.4 mA, 0.2 ms, 20s) on mesenteric blood flow (MBF) responses in a urethane-anesthetized male Sprague Dawley (SD) rats (n=7–9). Results are expressed as mean ± SEM.

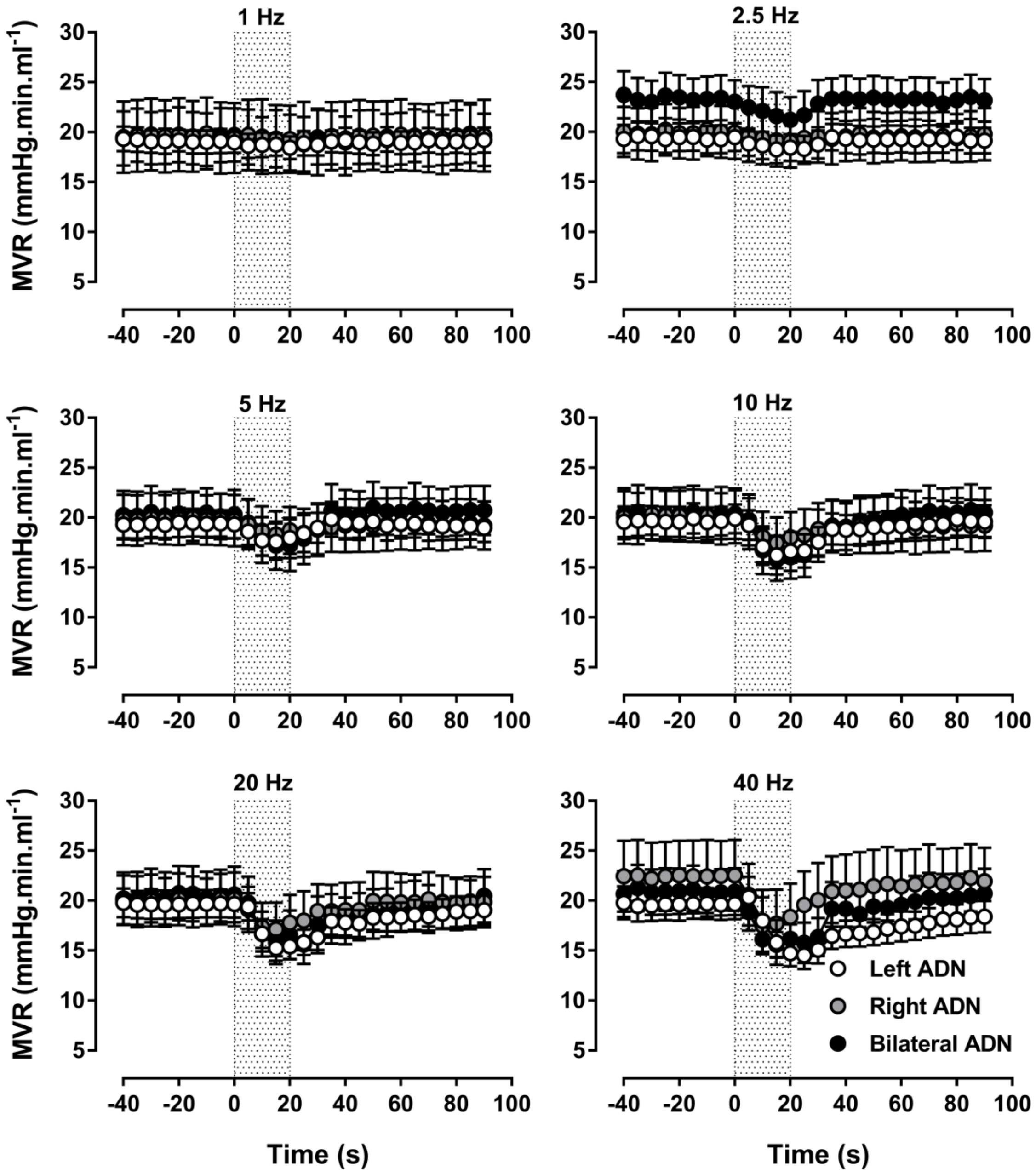

FIGURE S4: Time trend profile for the effects of left, right and bilateral aortic depressor nerve (ADN) stimulation (1–40 Hz, 0.4 mA, 0.2 ms, 20s) on mesenteric vascular resistance (MVR) responses in a urethane-anesthetized male Sprague Dawley (SD) rats (*n*=7–9). Results are expressed as mean ± SEM.

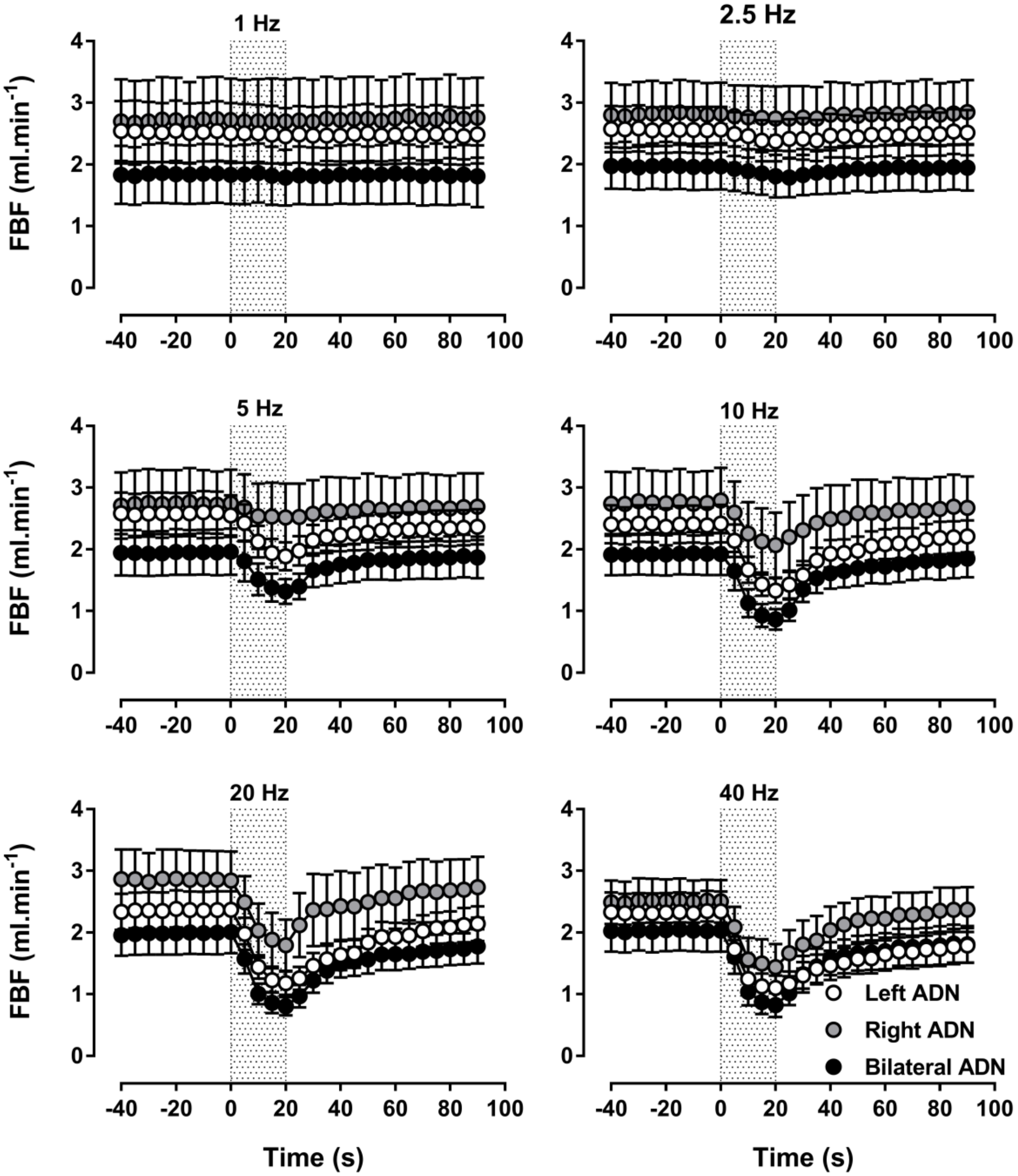

FIGURE S5: Time trend profile for the effects of left, right and bilateral aortic depressor nerve (ADN) stimulation (1–40 Hz, 0.4 mA, 0.2 ms, 20s) on femoral blood flow (FBF) responses in a urethane-anesthetized male Sprague Dawley (SD) rats (*n*=9–11). Results are expressed as mean ± SEM.

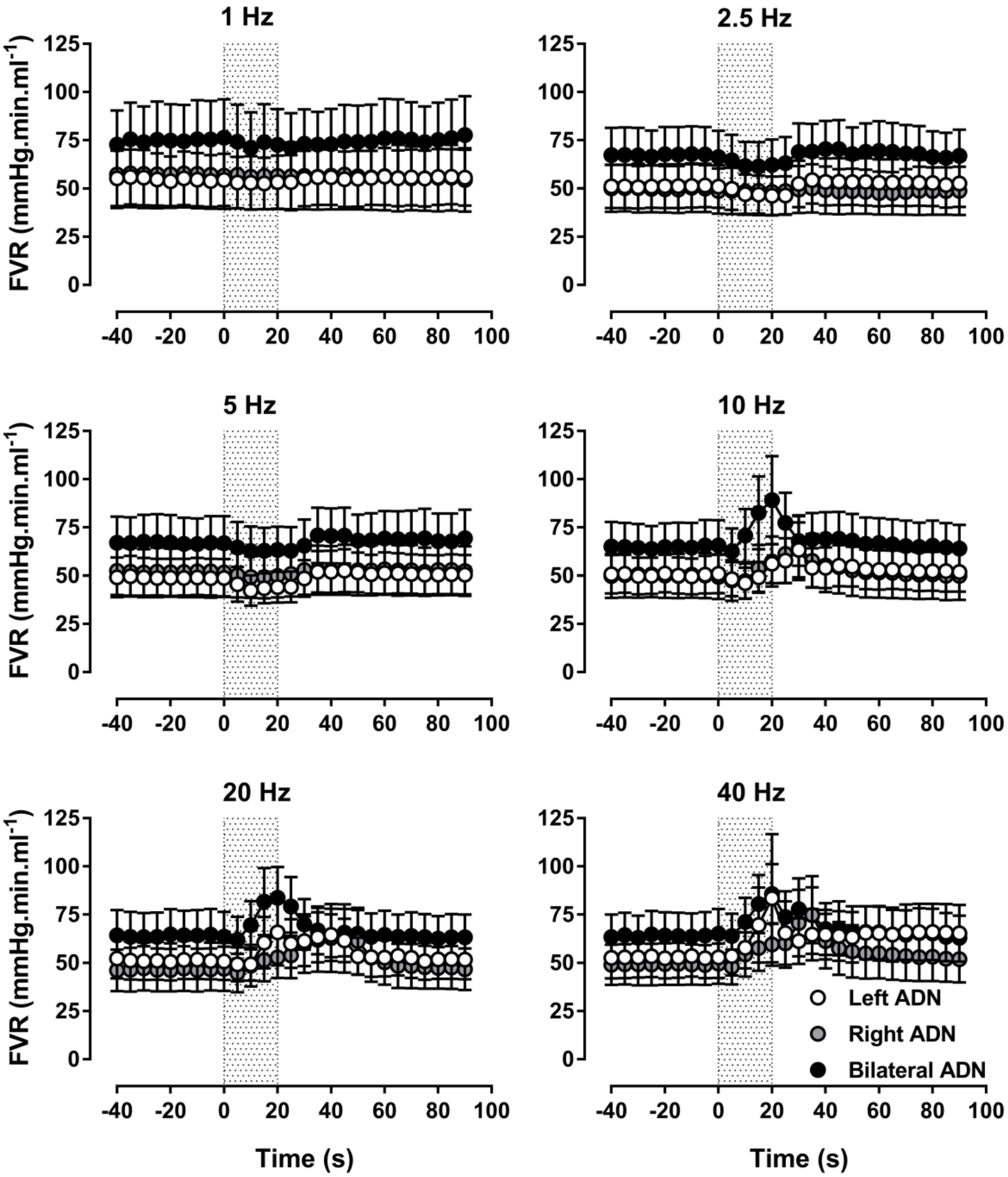

FIGURE S6: Time trend profile for the effects of left, right and bilateral aortic depressor nerve (ADN) stimulation (1–40 Hz, 0.4 mA, 0.2 ms, 20s) on femoral vascular resistance (FVR) responses in a urethane-anesthetized male Sprague Dawley (SD) rats (*n*=9–11). Results are expressed as mean ± SEM.

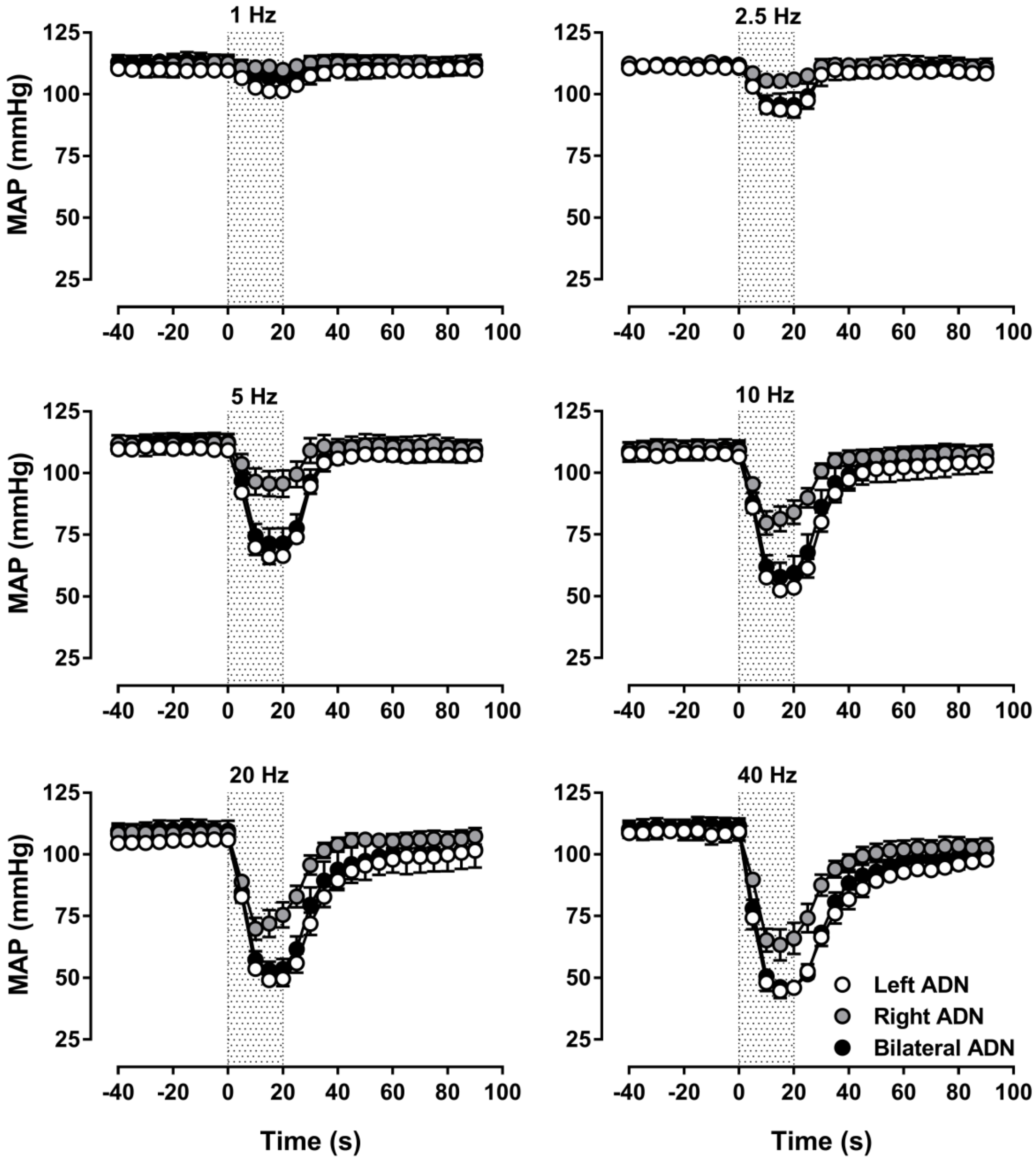

FIGURE S7: Time trend profile for the effects of left, right and bilateral aortic depressor nerve (ADN) stimulation (1–40 Hz, 0.4 mA, 0.2 ms, 20s) on mean arterial pressure (MAP) responses in a urethane-anesthetized female Sprague Dawley (SD) rats (*n*=7). Results are expressed as mean ± SEM.

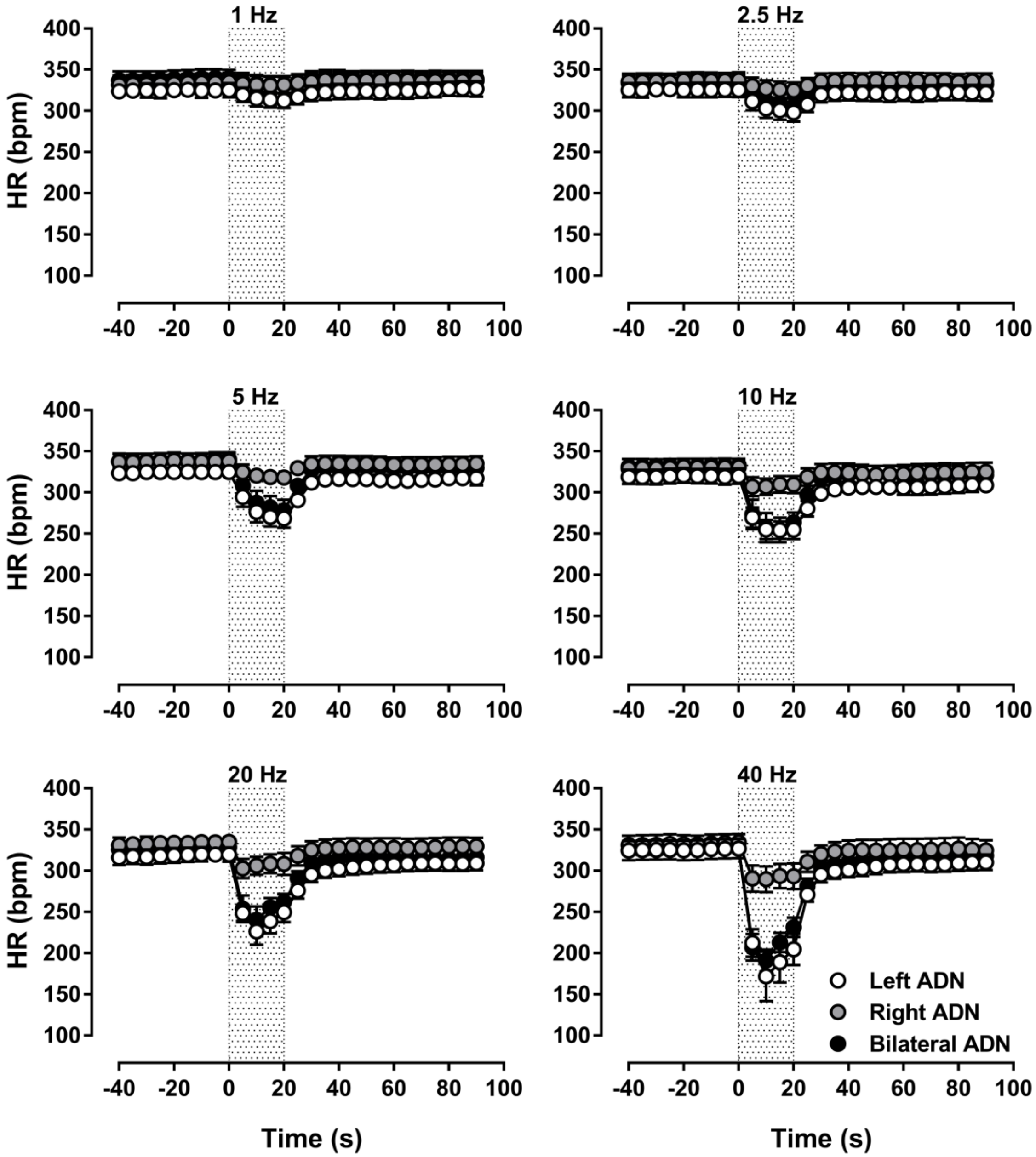

FIGURE S8: Time trend profile for the effects of left, right and bilateral aortic depressor nerve (ADN) stimulation (1–40 Hz, 0.4 mA, 0.2 ms, 20s) on heart rate (HR) responses in a urethane-anesthetized female Sprague Dawley (SD) rats (*n*=7). Results are expressed as mean ± SEM.

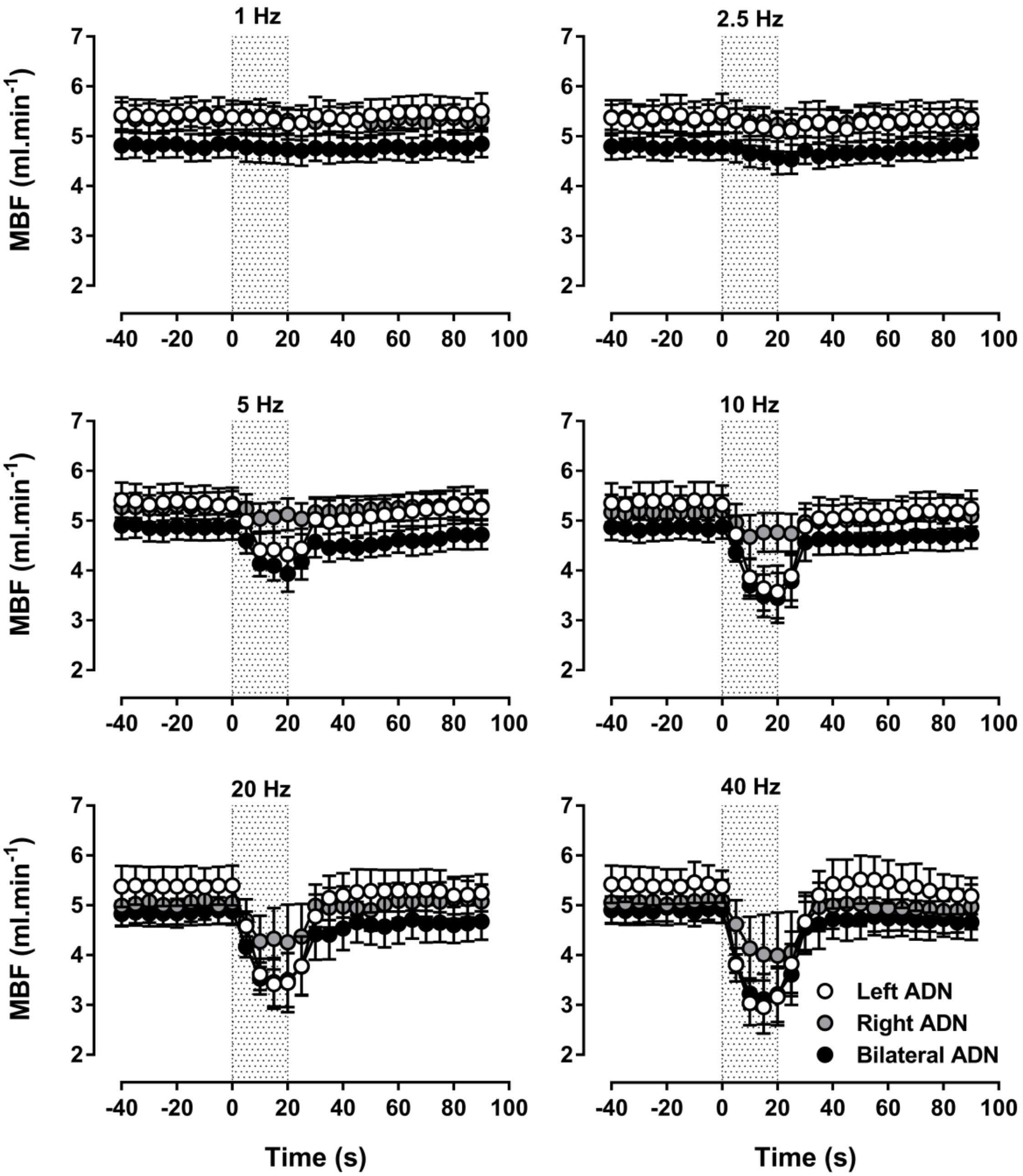

FIGURE S9: Time trend profile for the effects of left, right and bilateral aortic depressor nerve (ADN) stimulation (1–40 Hz, 0.4 mA, 0.2 ms, 20s) on mesenteric blood flow (MBF) responses in a urethane-anesthetized female Sprague Dawley (SD) rats (*n*=6–7). Results are expressed as mean ± SEM.

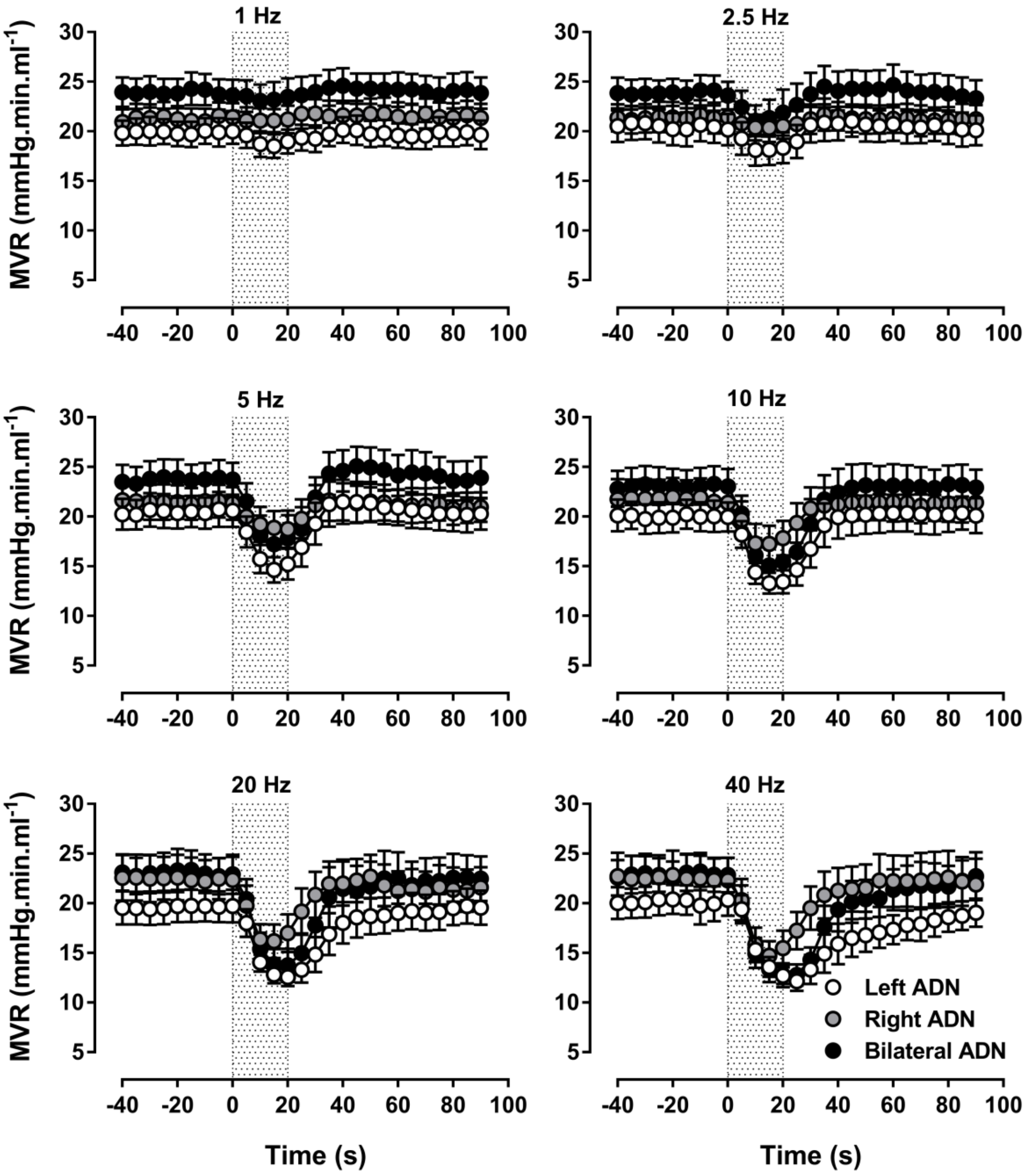

FIGURE S10: Time trend profile for the effects of left, right and bilateral aortic depressor nerve (ADN) stimulation (1–40 Hz, 0.4 mA, 0.2 ms, 20s) on mesenteric vascular resistance (MVR) responses in a urethane-anesthetized female Sprague Dawley (SD) rats (*n*=6–7). Results are expressed as mean ± SEM.

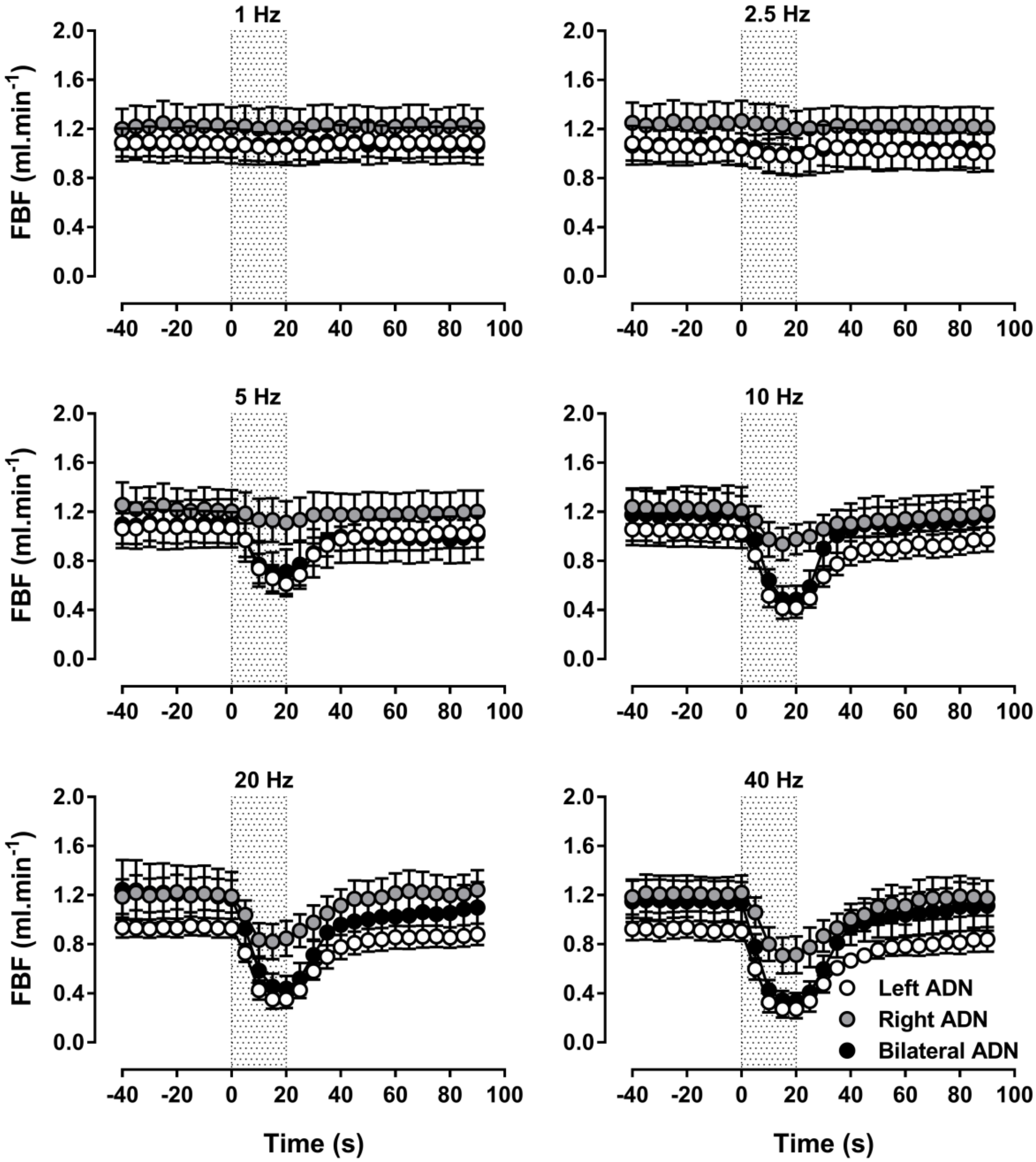

FIGURE S11: Time trend profile for the effects of left, right and bilateral aortic depressor nerve (ADN) stimulation (1–40 Hz, 0.4 mA, 0.2 ms, 20s) on femoral blood flow (FBF) responses in a urethane-anesthetized female Sprague Dawley (SD) rats (*n*=7). Results are expressed as mean ± SEM.

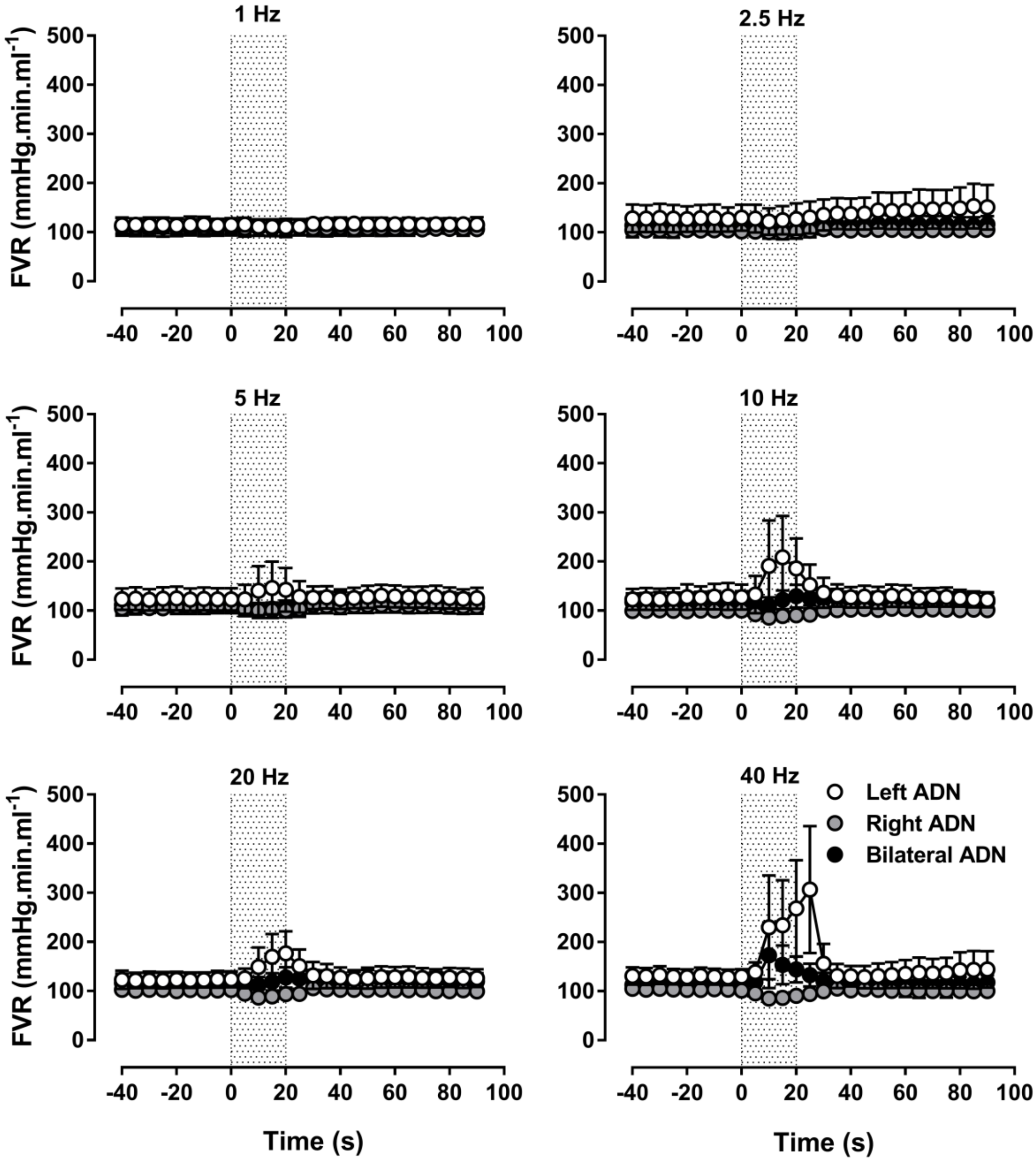

FIGURES 12: Time trend profile for the effects of left, right and bilateral aortic depressor nerve (ADN) stimulation (1–40 Hz, 0.4 mA, 0.2 ms, 20s) on femoral vascular resistance (FVR) responses in a urethane-anesthetized female Sprague Dawley (SD) rats (*n*=6–7). Results are expressed as mean ± SEM.

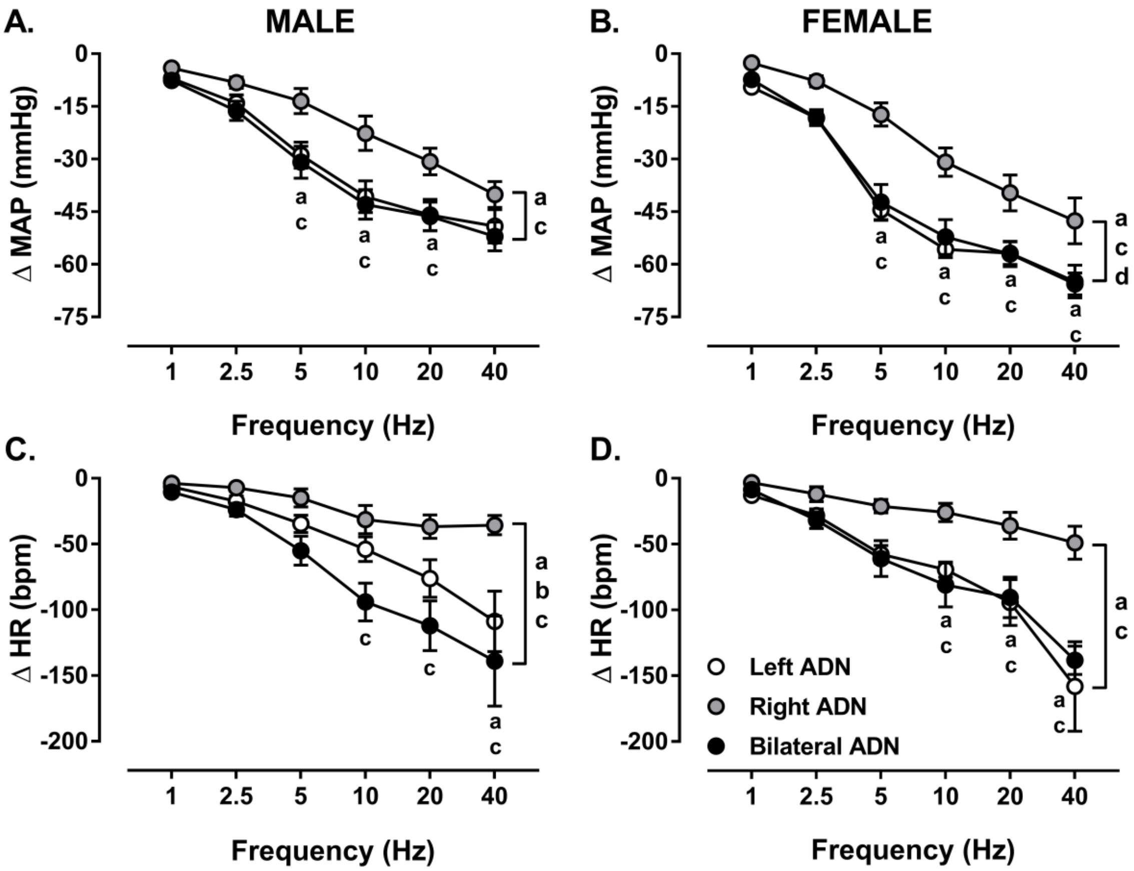

FIGURE S13: Effects of left, right and bilateral aortic depressor nerve (ADN) stimulation (1–40 Hz, 0.4 mA, 0.2 ms, 20s) on mean arterial pressure (MAP) **(A,B)** and heart rate (HR) **(C,D)** responses in urethane-anesthetized male **(left panels)** and female **(right panels)** Sprague Dawley (SD) rats (*n*=7–11). Results are reported as absolute changes and expressed as mean ± SEM. [a] $P \leq 0.05$, left vs. right ADN, [c] $P \leq 0.05$, right vs. bilateral ADN and [d] $P \leq 0.05$, female vs. male for respective left and bilateral ADN analyzed by a two-way ANOVA followed by Bonferroni's post-hoc.

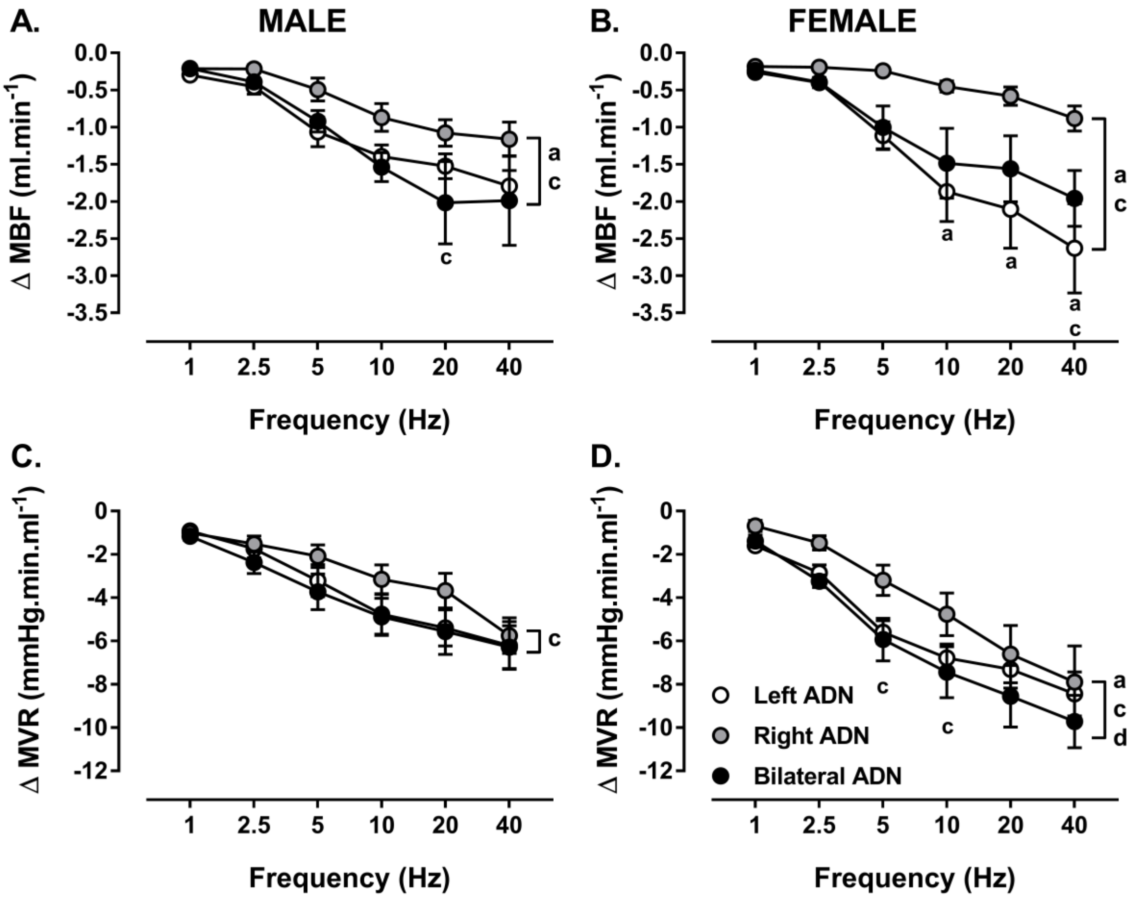

FIGURE S14: Effects of left, right and bilateral aortic depressor nerve (ADN) stimulation (1–40 Hz, 0.4 mA, 0.2 ms, 20s) on mesenteric blood flow (MBF) **(A,B)** and mesenteric vascular resistance (MVR) **(C,D)** responses in urethane-anesthetized male **(left panels)** and female **(right panels)** Sprague Dawley (SD) rats (*n*=6–9). Results are reported as absolute changes and expressed as mean ± SEM. [a] *P*≤0.05, left vs. right ADN, [c] *P*≤0.05, right vs. bilateral ADN and [d] *P*≤0.05, female vs. male for respective left and bilateral ADN analyzed by a two-way ANOVA followed by Bonferroni's post-hoc.

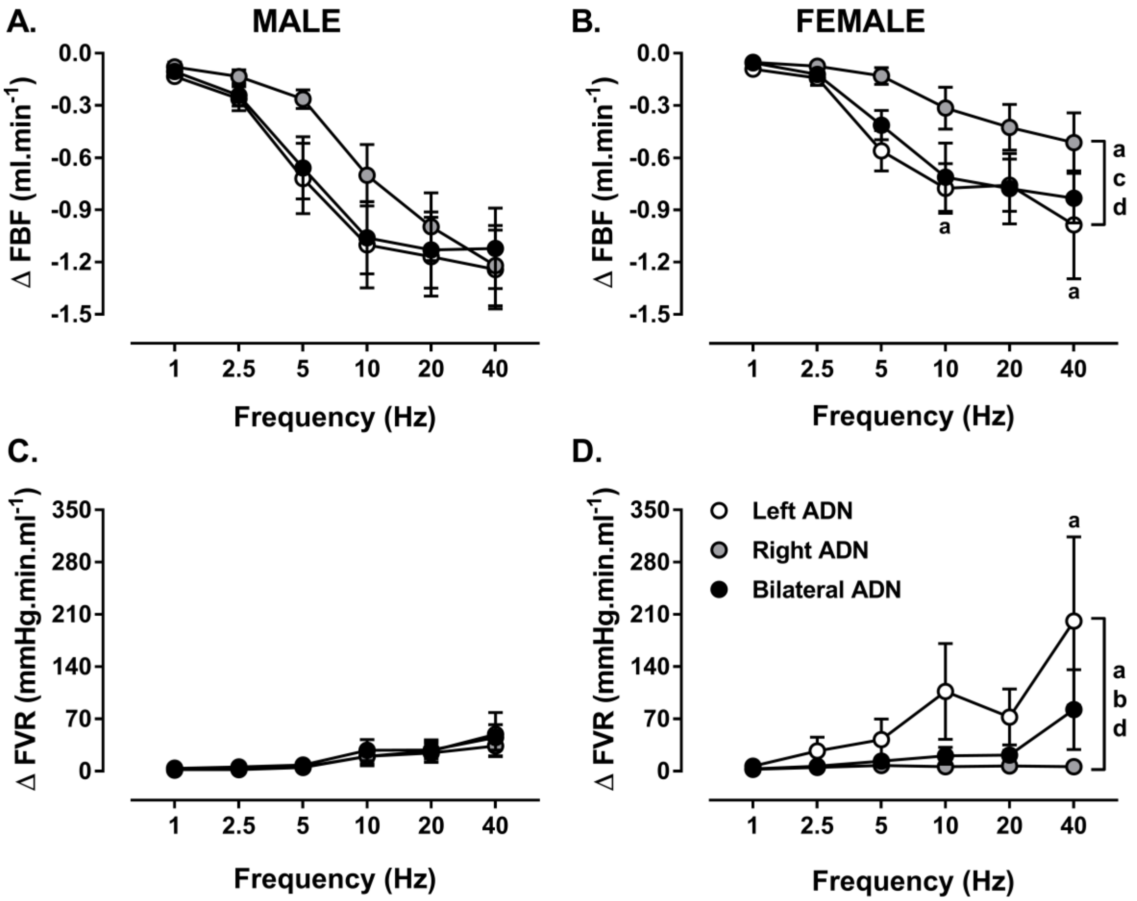

FIGURE S15: Effects of left, right and bilateral aortic depressor nerve (ADN) stimulation (1–40 Hz, 0.4 mA, 0.2 ms, 20s) on femoral blood flow (FBF) **(A,B)** and femoral vascular resistance (FVR) **(C,D)** responses in urethane-anesthetized male **(left panels)** and female **(right panels)** Sprague Dawley (SD) rats (n=7–11). Results are reported as absolute changes and expressed as mean ± SEM. [a] $P \leq 0.05$, left vs. right ADN, [b] $P \leq 0.05$, left vs. bilateral ADN, [c] $P \leq 0.05$, right vs. bilateral ADN and [d] $P \leq 0.05$, female vs. male for respective right ADN (Fig. A & B) and for respective left ADN (Fig. C & D) analyzed by a two-way ANOVA followed by Bonferroni's post-hoc.
